# Diagnostic significance of circulating miRNAs in systemic lupus erythematosus

**DOI:** 10.1371/journal.pone.0217523

**Published:** 2019-06-04

**Authors:** Xiaolan Zheng, Yi Zhang, Peng Yue, Lei Liu, Chuan Wang, Kaiyu Zhou, Yimin Hua, Gang Wu, Yifei Li

**Affiliations:** 1 Department of Pediatrics, West China Second University Hospital, Sichuan University, Chengdu, Sichuan, China; 2 Ministry of Education Key Laboratory of Women and Children's Diseases and Birth Defects, West China Second University Hospital, Sichuan University, Chengdu, Sichuan, China; 3 West China Medical School, Sichuan University, Chengdu, Sichuan, China; Chulalongkorn University, THAILAND

## Abstract

**Background:**

In recent years, many studies focused on the association between the microRNAs (miRNAs) and the risk of systemic lupus erythematosus (SLE), especially miRNA-21 (miR-21). We aimed to investigate the role of circulating miRNAs, especially the miR-21, as a biomarker in detecting SLE.

**Methods:**

We searched PubMed, EMBASE, the Cochrane Central Register of Controlled Trials, and China National Knowledge Infrastructure through Mar 3th, 2019. We performed this meta-analysis in a fixed/random-effect model using Meta-disc 1.4 and STATA 15.1.

**Results:**

A total of 17 relevant studies were eligible to analyze pooled accuracy. The overall performance of total mixed miRNAs (TmiRs) detection was: pooled sensitivity, 0.71 (95% confidence interval [CI], 0.69 to 0.72); pooled specificity, 0.81 (95%CI, 0.79 to 0.83); and area under the summary receiver operating characteristic curves value (SROC), 0.8797. The miR-21 detection was: pooled sensitivity, 0.68 (95%CI, 0.62 to 0.74); pooled specificity, 0.77 (95%CI, 0.69 to 0.84); and SROC, 0.8281. The meta-regression analysis showed that the type of samples was the sources of heterogeneity. The subgroup analysis suggested that detection in plasma group had the largest AUC of SROC in all the subgroups: pooled sensitivity, 0.8 (95%CI, 0.78 to 0.82); pooled specificity, 0.83 (95%CI, 0.8 to 0.86); and SROC, 0.9068.

**Conclusions:**

Our meta-analysis demonstrated that circulating miRNAs might be potential novel biomarkers for detecting SLE, especially miR-21. Moreover, plasma is recommended as the clinical specimen for diagnostic detection.

## Introduction

Systemic lupus erythematosus (SLE) is a complex, chronic, potentially fatal, multisystem autoimmune disease, which predominantly affects women between puberty and menopause [1]. The major pathogenetic mechanisms of SLE include an inappropriate immune response to the nucleic acid containing cellular particles, which is caused by an autoimmune reaction of the innate and adaptive immune systems, leading to damage structures of the skin, joints, kidney and central nervous system [2, 3]. Mortality in SLE patients has improved over the past 30 years but remains considerably higher than in people from the same geographical area without SLE [4]. Delay in diagnosis is associated with increased damage to vital organs [5]. In clinical practice, the diagnosis of SLE is mainly according to the revised American College of Rheumatology (ACR) classification criteria, which is made based on clinical manifestations and laboratory tests [6]. Laboratory tests, such as Complement (C3, C4), and antibodies (ANA, dsDNA, ACL, etc.) have although been demonstrated have potential as SLE diagnostic biomarkers, but none of them can accurately diagnose SLE [7–10]. The diagnosis of SLE is very challenging because there are no generally accepted diagnostic criteria [2]. Therefore, it is important to find novel and reliable circulating biomarkers for the diagnosis of SLE.

MicroRNAs (miRNAs) are a class of endogenously small noncoding RNAs with approximately 18–25 nucleotides in length [11], and may inhibit protein translation or degrading the polypeptides via binding to the untranslated regions of mRNA [12]. Previous studies [13–15] have found that circulating miRNAs can be considered as potential biomarkers for detecting kinds of diseases, including autoimmune diseases. In 2011, Stagaki et al. [16] suggested that miRNA-21 (miR-21) was a potential biomarker for SLE. Since then, a series of studies [17–21] have also verified the role of miR-21 in SLE. In addition, these studies [18–21] have found other miRNAs (miR-155, -181a, -196a, -31, and -148a) dysregulated in SLE. Dai et al [22] carried out a meta-analysis to verify the diagnostic accuracy of miRNAs as potential biomarkers for SLE. However, many new studies focused on the association between the miRNAs and the risk of SLE were reported in recent years, especially the relationship between miR-21and SLE. Therefore, we collected all published case-control studies to gather evidence on how the diagnostic performance of miRNAs, especially miR-21, distinguished SLE.

## Materials and methods

### Study protocol

This analysis was performed by a predetermined protocol following the recommendations of Deeks [23]. The data collection and reporting accorded with the Preferred Reporting Items for Systematic Reviews and Meta-Analyses (PRISMA) Statement (S1 Table) [24]. The ethical approval was not necessary due to it is systematic literature research.

### Search strategy

We searched multiple databases including PubMed, EMBASE, the Cochrane Central Register of Controlled Trials, and China National Knowledge Infrastructure through Mar 3th, 2019 to identify relevant studies. Keyword search terms were (‘systemic lupus erythematosus’ OR ‘lupus erythematosus’ OR ‘lupus nephritis‘) AND (‘MicroRNAs’ OR ‘MicroRNA’ OR ‘miRNAs’ OR ‘miRNA’). PubMed database was searched as follows: (Lupus Erythematosus, Systemic[MeSH Terms] OR systemic lupus erythematosus OR lupus erythematosus OR lupus nephritis) AND (MicroRNAs[MeSH Terms] OR MicroRNA OR miRNAs OR miRNA). Search terms for EMBASE, the Cochrane Central Register of Controlled Trials, and China National Knowledge Infrastructure with corresponding publication numbers can be found in the S1 Appendix. Language was limited only in English.

### Study selection

Reports were preliminarily screened by title and abstract and when initially selected by the systematic search. Potentially relevant studies were then retrieved by full manuscripts and assessed for compliance with inclusion and exclusion criteria.

Criteria for inclusion: (1) all patients of SLE were confirmed by SLE diagnosis criteria; (2) randomized controlled or non- randomized controlled, clinical trials, cohort studies evaluating the expression of miRNAs; (3) contained the data of true positive, false positive, false negative, and true negative; or the data of the receiver operating characteristic (ROC) curve, and essential sample size; (4) all studies had healthy controls; (5) real-time PCR (RT-PCR), qRT-PCR, microarray, and miRNA sequence were acceptable methods to evaluate the expressions of miRNAs; (6) full text published in English.

Criteria for Exclusion: (1) patients with malignant tumors or other autoimmune diseases; (2) conferences articles, reviews, letters, or case reports without controls; (3) no available data to construct a 2×2 table; (4) duplicated reports.

### Data collection and assessment of study quality

Two investigators (Xiaolan Zheng, Yi Zhang) screened and assessed the eligibility of reports at the title and/or abstract level independently according to the inclusion and exclusion criteria., and a third reviewer (Yifei Li) determining the divergences according to inclusion or exclusion criteria, and the quality of reports; studies that met all the inclusion criteria were selected for further analysis. According to the 14-item Quality Assessment of Diagnostic Accuracy Studies (QUADAS) list [25], the quality assessment of all enrolled studies was independently conducted by two investigators (Xiaolan Zheng, Peng Yue), and any disagreement was settled by discussion. As a well-conducted study might score poorly once related parts were missing among the methods and results so that all the assessments were only reported in descriptive forms. We used Photoshop CS6 (Adobe Systems Software Ireland Ltd) to extract data from the figures. By Photoshop CS6, we were able to set axes and identify the abscissa and ordinate of each point. Finally, two investigators (Xiaolan Zheng, Lei Liu) extracted the date which can calculate true positive, false positive, false negative, and true negative, such as sensitivity, specificity, and essential sample size.

### Evaluation indicators

The following indicators of different types of miRNAs were measured: sensitivity, specificity, diagnostic odds ratio (DOR), and area under the summary receiver operating characteristic curves value (SROC). Sensitivity was represented by the proportion of patients with SLE that was correctly identified by the positive results of miRNAs expression. Specificity was represented by the non-SLE cases that were correctly identified by the negative results of miRNAs. DOR was an independent indicator from 0 to infinity, indicating that patients with positive test results are much more likely to have SLE than patients with negative test results. The higher the DOR, the better the discriminatory ability of the test was [26]. The SROC was plotted according to the combination of sensitivity and specificity, and the area under the curve (AUC) value was then calculated as a global measurement of test performance. The closer the AUC was to 1, the better the test performance [27].

### Publication bias

We used Stata statistical software (STATA, version 15.1) to obtain a quantitative analysis of all the publication bias according to funnel plots and the Deek’s test. An asymmetric distribution of data points in the funnel plot with a quantified result of P < .05 indicated the presence of potential publication bias [28].

### Heterogeneity and meta-regression

Heterogeneity of pooling sensitivity and specificity was examined by the x^2^ test. Heterogeneity of pooling DOR was examined by the Cochran Q test. Heterogeneity was considered as statistically significant when P < .05. The I^2^ test was also performed in every pooling analysis in order to quantitatively estimate the proportion of total variation across studies, which was attributable to heterogeneity rather than chance. The I^2^ value would range from 0 to 100%, with values of 25, 50, and 75% considered as evidence of low, moderate, and high heterogeneity, respectively [29]. A curvilinear shape in the SROCs indicated the presence of a threshold effect. Furthermore, we carried out the meta-regression analysis using STATA 15.1 to detect where the potential factor for heterogeneity origin from.

### Sensitivity analysis and subgroup analysis

Sensitivity analysis was conducted for every study to determine the influence of individual trials on the results, using STATA 15.1 for meta-analysis fixed/random-effects estimates. Meta-Disc 1.4 was used to detect threshold effects in studies and conduct subgroup analysis.

### Statistical analysis

Data analysis and threshold analysis was performed with Meta-Disc Version 1.4 [30]. Besides, publication bias was conducted by STATA Version 15.1 (Stata Corporation, College Station, Texas, USA). Homogenous results utilized the fixed effects model for statistical analysis, while the heterogeneous (I^2^ > 50%) results utilized random effects model and the data were presented using a forest map.

## Results

### Search results

**Fig 1:**
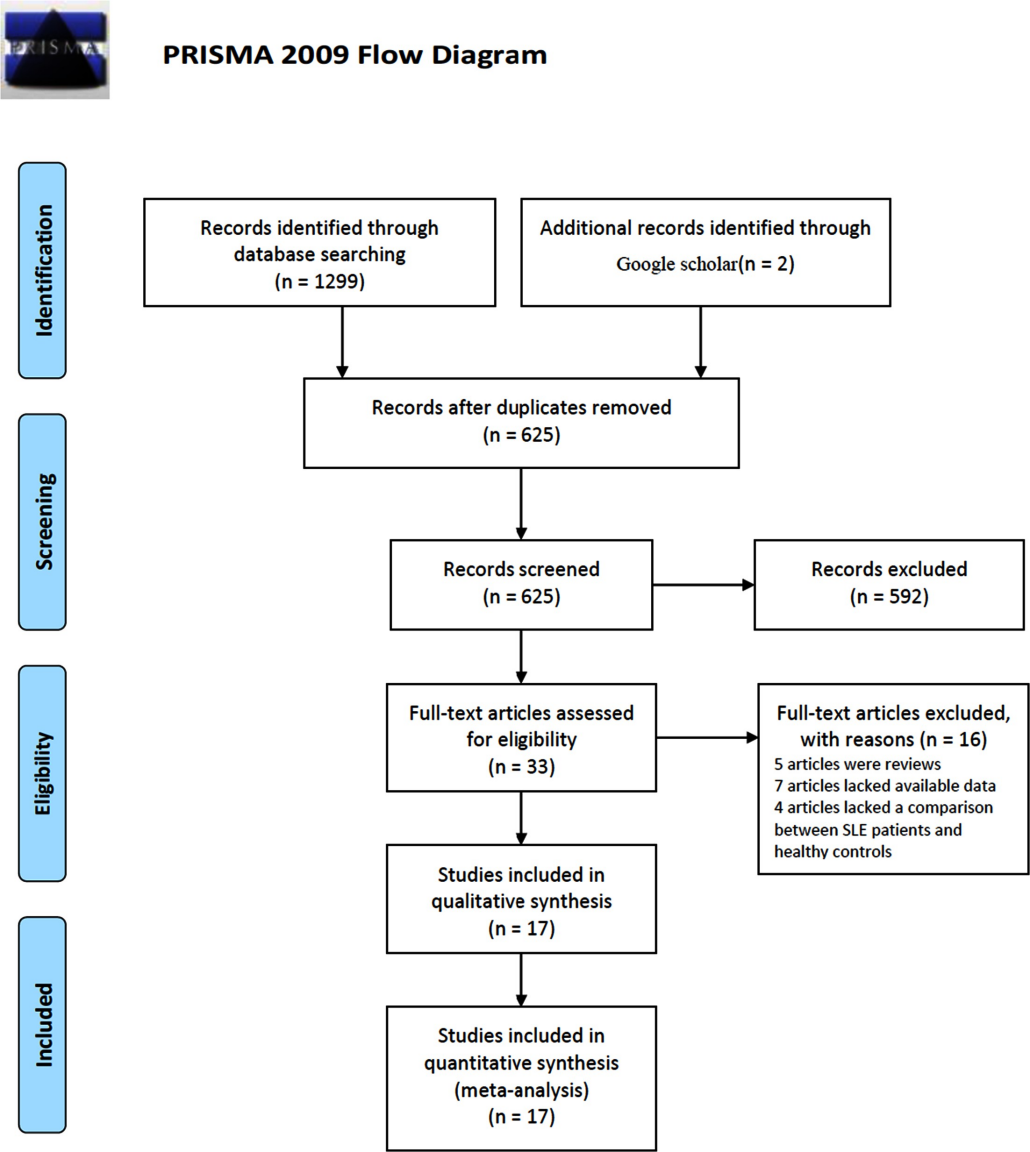
Flow diagram of the study selection process. From: Moher D, Liberati A, Tetzlaff J, Altman DG, The PRISMA Group (2009). Preferred Reporting Items for Systematic Reviews and Meta-Analyses: The PRISMA Statement. PLoS Med. 6(7): e1000097. doi: 10.1371/journal.pmed1000097. For more information, visit www.prisma-statement.org.

Initially, 1301 potentially relevant papers were retrieved by the search method aforementioned, of which 33 articles were considered to be interested after reading titles and abstracts. However, five articles were excluded by reading their complete articles due to article types, seven studies lacked available data to construct a 2×2 table, and four articles lacked a comparison between SLE patients and healthy controls. Finally, 17 studies [17–21, 31–42] were included in the meta-analysis. The process of study selection was illustrated in Fig 1. Except the study of Zununi Vahed et al. [42] is a cross-sectional trial, all others are prospective trials. Among them, 41 individual diagnostic tests were extracted for total mixed miRNA (TmiR) evaluation; five individual diagnostic tests were extracted for miR-21 evaluation, 36 individual diagnostic tests were enrolled for total mixed miRNA knock out miR-21 (TmiRs-KO-21) evaluation. Additionally, the sample types of nine studies were plasma, five studies were peripheral blood mononuclear cells (PBMC), and the remaining three studies were serum. Moreover, there are ten reports from China, in which ethnicity can be defined as Mongoloid; and seven studies from other different countries (two from Iran, one from Bulgaria, three from Egypt, and one from Colombia), in which ethnicity can be defined as Caucasian. Besides, the SLE sample size of 13 studies were much smaller (n < 100) compared the remaining four (n ≥ 100). The basic characteristics of the included studies was shown in Table 1.

**Table 1:** Characteristics of studies in meta-analysis.

No.	First author	Year	Countries	Ethnicity	Type of control	Golden standard	Detection method	Specimen	Case (male/female)	Age of case	Control (male/female)	Age of control	Selected miRNAs
1	Zununi Vahed S	2018	Iran	Caucasian	HC	ACR	qRT-PCR	plasma	26(6/20)	32.61	26(9/17)	29.9	miR-155, miR-125, miR-146, miR-142-3p
2	Khoshmirsafa M	2018	Iran	Caucasian	HC	ACR	qRT-PCR	PBMC	55(0/55)	36.7	30(0/30)	34.9	miR-155, miR-141, miR-21, miR-16
3	Shumnalieva R	2018	Bulgaria	Caucasian	HC	ACR	qRT-PCR	PBMC	40(0/40)	43.6	32(10/22)	39.15	miR-155, miR-146a
4	Zhang Y	2018	China	Mongoloid	HC	ACR	RT-PCR	plasma	101(16/85)	34.2	100(19/81)	33.3	miR-200b-5p, miR-141-5p, miR-200c-5p
5	Guo G	2018	China	Mongoloid	HC	ACR	qRT-PCR	PBMC	25(2/23)	28.63	25(4/21)	27.98	NovelmiRNA-25, miR-1273h-5p
6	Wang X	2018	China	Mongoloid	HC	ACR	qRT-PCR	PBMC	42(6/36)	32.12	35(5/30)	32.12	miR-29b
7	Zeng L	2018	China	Mongoloid	HC	ACR	qRT-PCR	serum	1002(N/R)	N/R	508(N/R)	34.5	miR-371b-5p, miR-5100
8	Zhang H	2018	China	Mongoloid	HC	ACR	qRT-PCR	plasma	50(10/40)	37.18	20(6/14)	N/R	miR-150, miR-92a, miR-27a, miR-19b, miR-25, miR-23a, miR-93, miR-181a, miR-22, miR-223, miR-16, miR-20a, miR-15b, miR-103
9	Zhu Y	2017	China	Mongoloid	HC	N/R	qRT-PCR	PBMC	128(16/112)	40.6	30(4/26)	39.3	miR-146a
10	Sharaf-Eldin WE	2017	Egypt	Caucasian	HC	ACR	qRT-PCR	serum	10	N/R	23	N/R	miR-145, miR-223, miR-326
11	Li HS	2017	China	Mongoloid	HC	ACR	qRT-PCR	plasma	100(22/78)	31.5	40	N/R	miR-181a, miR-203
12	Amr KS	2016	Egypt	Caucasian	HC	ACR	qRT-PCR	plasma	40(0/40)	30.3	20(0/20)	34	miR-31, miR-21
13	S Guo	2016	China	Mongoloid	HC	ACR	qRT-PCR	plasma	44(2/42)	35	24(3/21)	36	miR-21, miR-126, miR-148a
14	Motawi TK	2016	Egypt	Caucasian	HC	ACR	qRT-PCR	plasma	70(5/65)	30.06	30(5/25)	31.87	miR-21, miR-181a, miR-196a
15	Navarro-Quiroz E	2016	Colombia	Caucasian	HC	ISN/RPS	qRT-PCR	plasma	40	N/R	40	N/R	miR-221-5p, miR-380-3p, miR-556-5p, miR-758-3p, miR-3074-3p
16	Wang W	2015	China	Mongoloid	HC	ACR	qRT-PCR	serum	40(2/38)	42.03	32(2/30)	42.03	miR-130b-3p
17	Tang ZM	2014	China	Mongoloid	HC	ACR	qRT-PCR	plasma	44(4/40)	39	36(4/32)	38	miR-21

HC = healthy control, ACR = American College of Rheumatology, ISN/RPS = International Society of Nephrology/Renal Pathology Society, miR = mircoRNA, PBMC = peripheral blood mononuclear cell, N/R = not report.

### Study quality

The quality assessment of the included studies was accomplished by using the QUADAS list of questions, and the results were shown in Table 2.

**Table 2:** QUADAS criteria of included studies.

No.	Spectrum composition	Selection criteria	Reference standard	Disease progression bias	Partial verification	Differential verification	Incorporation bias	Index test execution	Reference standard execution	Test review bias	Reference standard review bias	Clinical review bias	Uninterruptible test results	Withdrawals
1	+	+	+	+	+	+	+	+	+	+	+	+	+	+
2	+	+	+	+	+	+	+	+	+	+	+	+	+	+
3	+	+	+	+	+	+	+	+	+	+	+	+	+	+
4	+	+	+	+	+	+	+	+	+	+	+	+	+	?
5	+	+	+	+	+	+	+	+	+	+	+	+	+	+
6	+	+	+	+	+	+	+	+	+	+	+	+	+	+
7	+	+	+	?	+	+	+	+	+	+	?	+	+	-
8	+	+	+	+	+	+	+	+	+	+	+	+	+	+
9	?	+	?	+	+	?	+	+	?	+	+	+	+	+
10	?	+	+	+	+	+	+	+	+	+	?	+	+	?
11	+	+	+	+	+	+	+	+	+	+	+	+	+	+
12	+	+	+	+	+	+	+	+	+	+	+	+	+	+
13	+	+	+	+	+	+	+	+	+	+	+	+	+	+
14	+	+	+	+	+	+	+	+	+	+	+	+	+	+
15	?	+	+	+	+	+	+	?	+	+	+	+	+	?
16	+	+	+	+	+	+	+	+	+	+	+	+	+	+
17	+	+	+	+	+	+	+	+	+	+	+	+	+	+

QUADAS = Quality Assessment of Diagnostic Accuracy Studies.

### Diagnostic accuracy of miRNAs

#### Total mixed miRNAs

**Fig 2:**
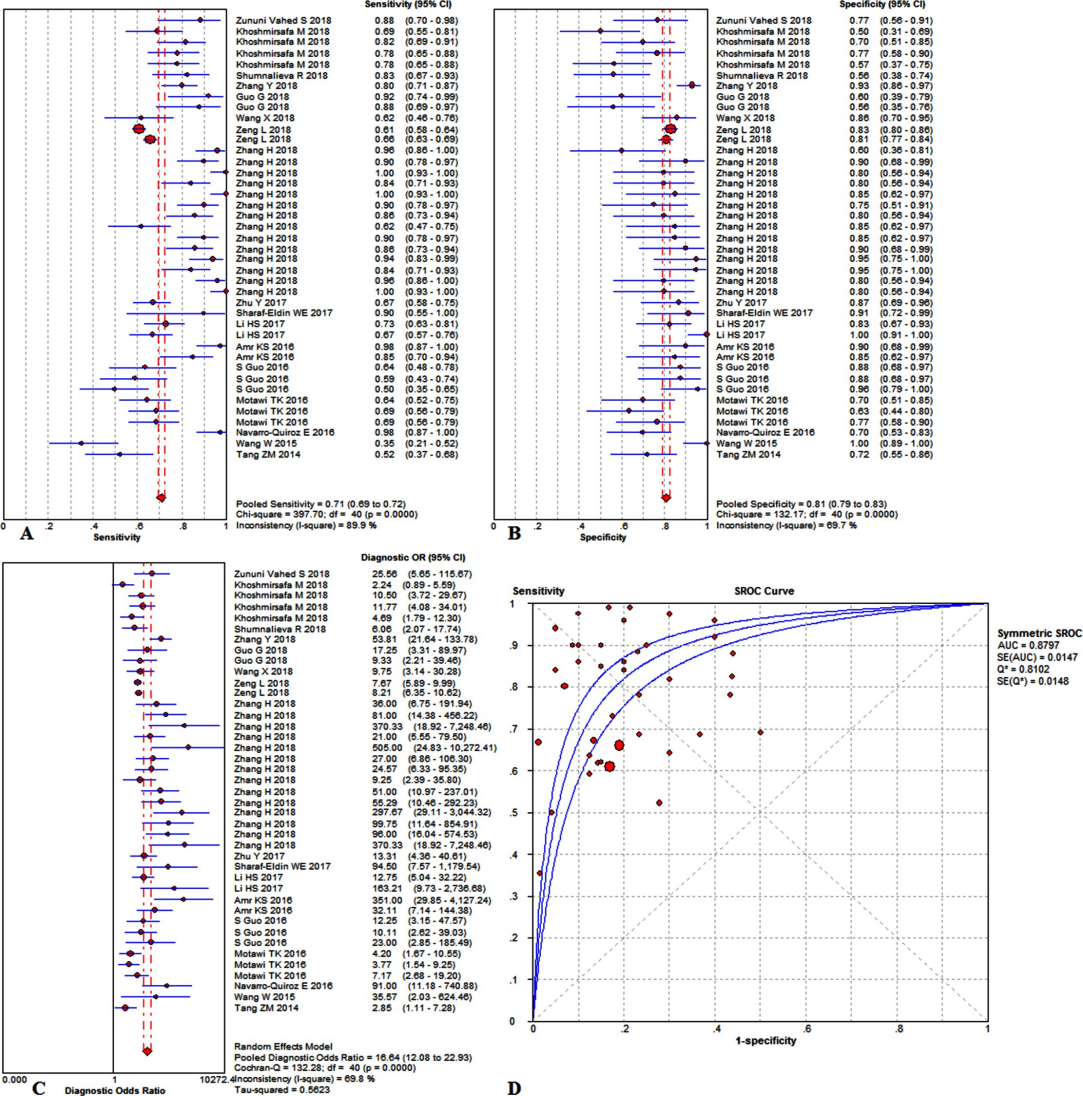
Performance of TmiRs detection for the diagnosis of SLE. (A) Pooled sensitivity. (B) Pooled specificity. (C) Overall DOR. (D) The SROCs for all datasets. The point estimates from each study are shown as solid squares. The pooled estimates are shown as a solid diamond. Effect sizes were pooled by random-effect models. Each square in the SROC represents 1 study. Sample size is indicated by the size of the square. Error bars represent 95% CIs. CI = confidence interval, DOR = diagnostic odds ratio, SLE = systemic lupus erythematosus, miR = mircoRNA, OR = odds ratio, SROC = summary receiver operating characteristic curves value.

**Fig 3:**
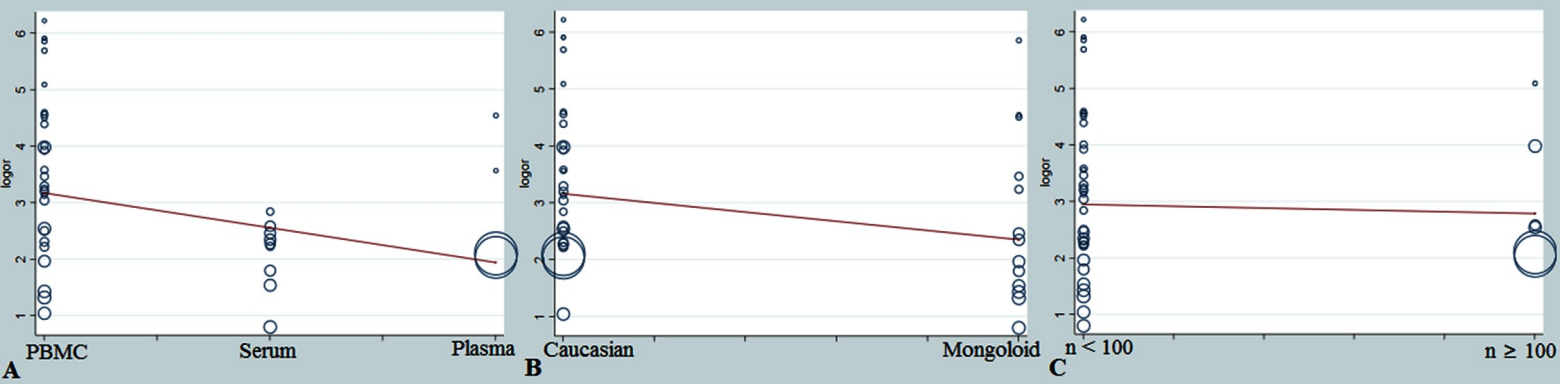
The meta-regression of the enrolled studies. (A) For the type of samples, the meta-regression detected it was a dramatic impact on the homogeneity of the enrolled studies, P = .030, t = -2.17, 95%CI (0.30, 0.96). (B) For the ethnicity of studies, the meta-regression did not find it was a dramatic impact on the homogeneity of the enrolled studies, P = .056, t = -1.97, 95%CI (0.20, 1.02). (C) For the SLE sample sizes, the meta-regression did not detect it was a dramatic impact on the homogeneity of the enrolled studies, P = .77, t = -0.30, 95%CI (0.29, 2.52). The meta-regression could determine the correlation between the potential factors and the existing heterogeneities. When a significant difference was discovered, the factor should have a dramatic impact on the homogeneity of the enrolled studies with a P value <0.05. or, odds ratio. CI, confidence interval.

The overall diagnostic measurement in detecting SLE of TmiRs has been summarized in Fig 2. The summary sensitivity was 0.71 (95%CI, 0.69 to 0.72), and the pooled estimation showed significant heterogeneity (P = .0000, x^2^ = 397.7, I^2^ = 89.9%) (Fig 2A). Meanwhile, the summary specificity was 0.81 (95%CI, 0.79 to 0.83), and the pooled estimation also showed noticeable heterogeneity (P = .0000, x^2^ = 132.17, I^2^ = 69.7%) (Fig 2B). In addition, the pooled DOR and the SROCs showed in Fig 2C and 2D. The pooled DOR was 16.64 (95% CI, 12.08 to 22.93) with significant heterogeneity (P = .0000, Cochran-Q = 132.28, I^2^ = 69.8%). The calculated AUC value was 0.8797±0.0147. The absence of a curvilinear shape in the SROC suggested no potential presence of a threshold effect. Next, we carried out the meta-regression analysis to identify the potential factors that might cause the heterogeneities. The meta-regression could determine the correlation between the potential factors and the existing heterogeneities. When a significant difference was discovered, the factor should have a dramatic impact on the homogeneity of the enrolled studies with a P value <0.05. After reviewing the baseline data and the original data producing procedure, the types of sample, ethnicity, and SLE sample sizes were taken into account in the meta-regression to detect the origins of heterogeneities. According to the results (Fig 3), the type of samples might be the sources of heterogeneity, P = .036, t = -2.17, 95%CI (0.30, 0.96) (Fig 3A). Besides, the ethnicity of studies is not a dramatic impact factor on the homogeneity of the enrolled studies, P = .056, t = -1.97, 95%CI (0.20, 1.02) (Fig 3B). Meanwhile, the meta-regression also found the SLE sample sizes is not a dramatic impact factor, P = .77, t = -0.30, 95%CI (0.29, 2.52) (Fig 3C). Therefore, the type of samples might be responsible for the existing heterogeneities.

#### miRNA-21

**Fig 4:**
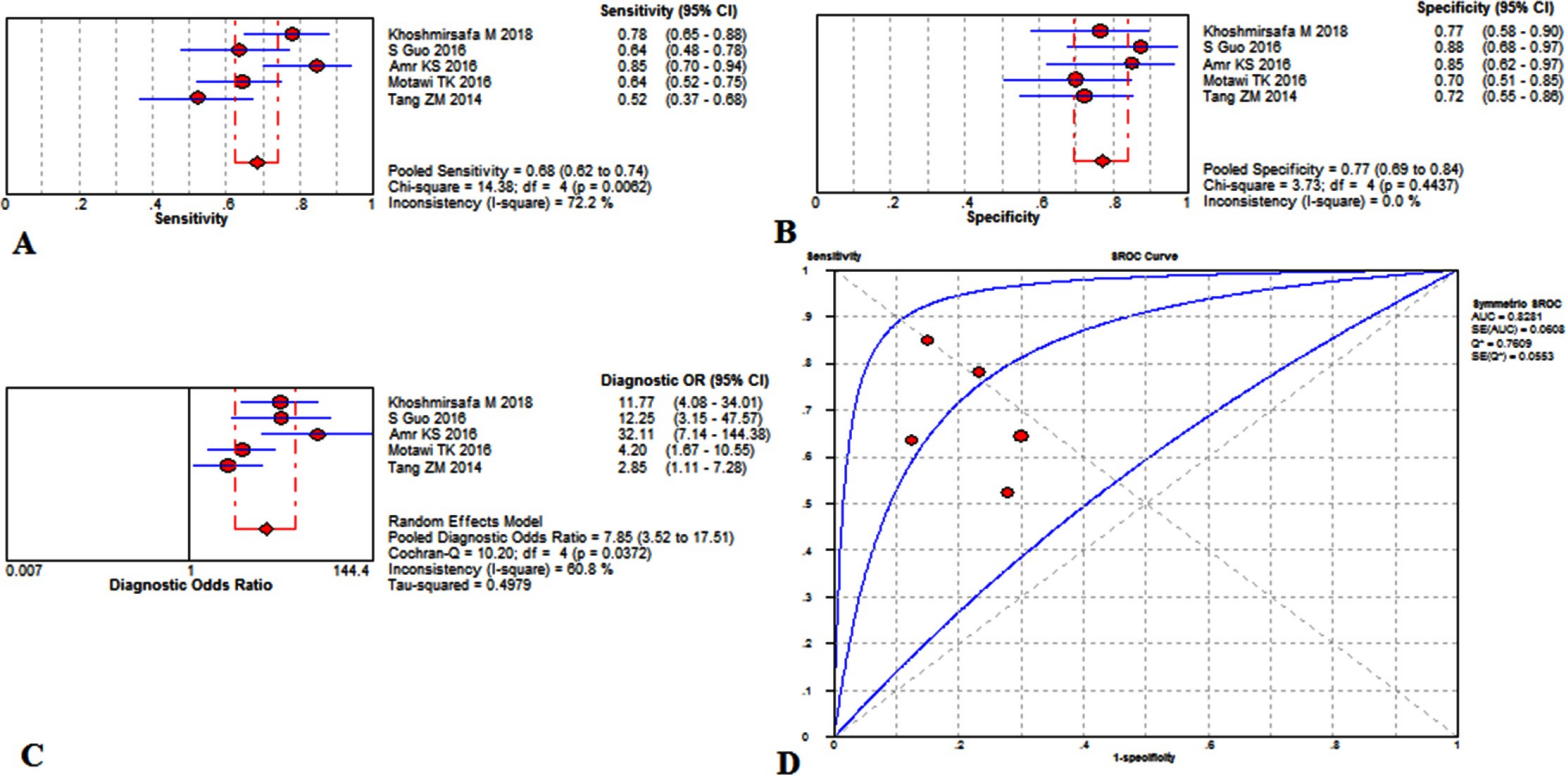
Performance of miR-21 detection for the diagnosis of SLE. (A) Pooled sensitivity. (B) Pooled specificity. (C) Overall DOR. (D) The SROCs for all datasets. The point estimates from each study are shown as solid squares. The pooled estimates are shown as a solid diamond. Effect sizes were pooled by random-effect models. Each square in the SROC represents 1 study. Sample size is indicated by the size of the square. Error bars represent 95% CIs. CI = confidence interval, DOR = diagnostic odds ratio, SLE = systemic lupus erythematosus, miR = mircoRNA, OR = odds ratio, SROC = summary receiver operating characteristic curves value.

The overall diagnostic performance in detecting SLE of miR-21 has been demonstrated in Fig 3. The summary sensitivity was 0.68 (95%CI, 0.62 to 0.74), and the pooled estimation showed significant heterogeneity (P = .0062, x^2^ = 14.38, I^2^ = 72.2%) (Fig 4A). Additionally, the summary specificity was 0.77 (95%CI, 0.69 to 0.84), and the pooled estimation showed some heterogeneity (sensitivity: P = .4437, x^2^ = 3.73, I^2^ = 0.0%) (Fig 4B). Meanwhile, the pooled DOR was 7.85 (95%CI, 3.52 to 17.51) with a noticeable heterogeneity (P = .0372, Cochran-Q = 10.20, I^2^ = 60.8%) (Fig 4C). It revealed that the AUC value was 0.8281±0.0608 (Fig 4D). The absence of a curvilinear shape in the SROC suggested no potential presence of a threshold effect. As fewer than ten studies were included in this study, meta-regression and subgroups analysis was not appropriate.

#### Total mixed miRNAs with miR-21 knockout

The overall diagnostic performance of TmiRs-KO-21 (S1 Fig) showed the potential diagnostic capability of miRNAs for SLE without the impact of miR-21. The summary sensitivity was 0.71 (95%CI, 0.69 to 0.72), and the summary specificity was 0.81 (95%CI, 0.79 to 0.83). Both pooled estimations showed high heterogeneity (sensitivity: P = .0000, x^2^ = 382.58, I^2^ = 90.9%; specificity: P = .0000, x^2^ = 127.14, I2 = 72.5%) (Figure A and B in S1 Fig). The pooled DOR was 19.06 (95%CI, 13.42 to 27.06). The results of DOR showed consistency across the included reports, with noticeable heterogeneity (P = .0000, Cochran-Q = 119.49, I^2^ = 70.7%) (Figure C in S1 Fig). The AUC value was 0.8895±0.0152 (Figure D in S1 Fig). The absence of a curvilinear shape in the SROC suggested no potential presence of a threshold effect.

#### Sensitivity analysis and subgroup analysis

**Fig 5:**
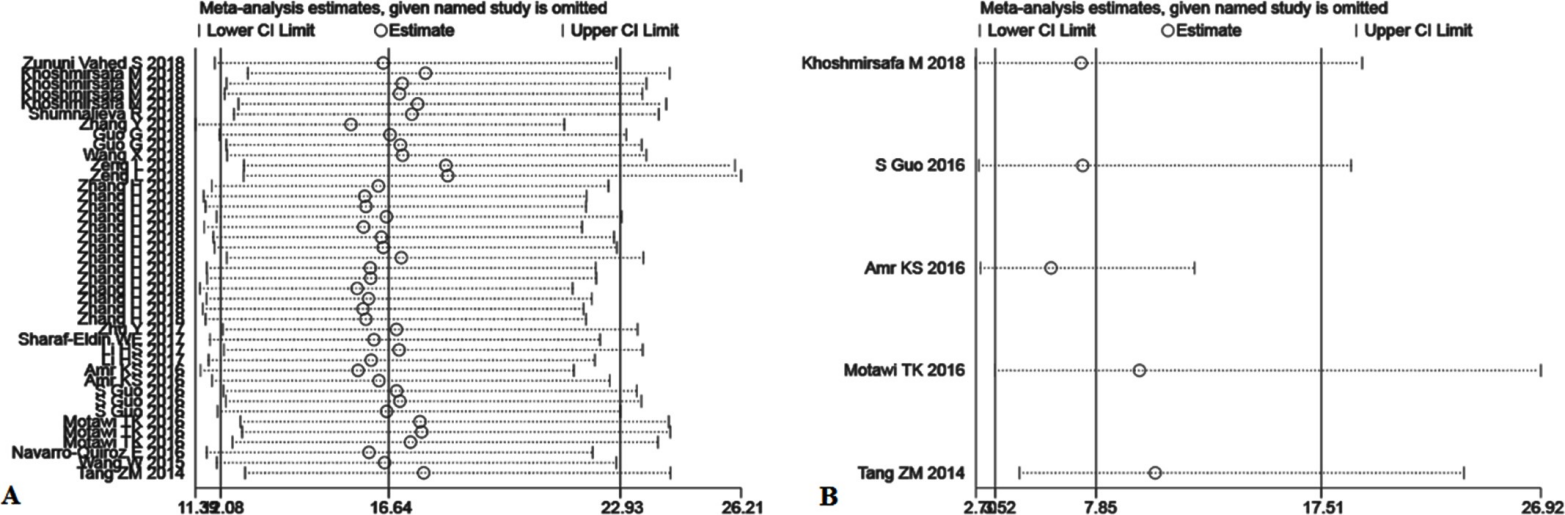
Sensitivity analysis of the individual trials on the results TmiRs and miR-21. (A) For the result of TmiRs, (B) For the result of miR-21. Not any single study was detected to incur undue weight in the analysis.

We systematically and qualitatively analyzed the sensitivity across included studies to determine the influence of individual trials on the results of TmiRs and miR-21, using STATA 15.1 for meta-analysis random-effects estimates. Finally, we did not detect any significant impact from every single research and confirmed the results of TmiRs (Fig 5A) and miR-21 (Fig 5B). Besides, we used Meta-Disc 1.4 to detect whether there is any threshold effect in studies. The results showed that the Spearman correlation coefficient was 0.178 and P = .264, meaning no threshold effect related to heterogeneity existed. Among the all enrolled studies, there were three different types of sample collection protocols (plasma, serum, and PBMC), two ethnicities (Caucasian and Mongoloid), and two SLE sample sizes (n < 100 and n ≥ 100). And then, we conducted three subgroups analysis according to the type of samples, ethnicity, and the SLE sample size. The results were shown in Table 3 and S2–S8 Figs. The subgroup analysis suggested that the plasma group had the largest AUC of SROC in all the subgroups: pooled sensitivity, 0.8 (95%CI, 0.78 to 0.82); pooled specificity, 0.83 (95%CI, 0.8 to 0.86); and SROC, 0.9068.

**Table 3:** Subgroup analysis results of included studies.

	Sensitivity (95% CI)	Specificity(95% CI)	DOR(95% CI)	SROC(AUC±SE)
TmiRs	0.71(0.69–0.72)	0.81(0.79–0.83)	16.64(12.08–22.93)	0.8797±0.0147
P/I^2^	.0000/89.9%	.0000/69.7%	.0000/69.8%	-
Type of samples				
PBMC	0.75(0.71–0.79)	0.67(0.61–0.73)	7.33(5.23–10.28)	0.8044±0.0279
P/I^2^	.0137/58.4%	.0040/64.5%	.1955/28.0%	-
Serum	0.63(0.61–0.65)	0.83(0.80–0.85)	8.17(6.87–9.71)	0.7973±0.0346
P/I^2^	.0001/86.5%	.0021/79.6%	.1833/38.1%	-
Plasma	0.8(0.78–0.82)	0.83(0.8–0.86)	28.38(16.96–47.50)	0.9068±0.0151
P/I^2^	.0000/88.2%	.0002/55.9%	.0000/70.3%	-
Ethnicity				
Caucasian	0.78(0.74–0.81)	0.70(0.65–0.75)	10.37(5.70–18.85)	0.8028±0.0457
P/I^2^	.0000/74.5%	.0102/54.1%	.0002/68.3%	-
Mongoloid	0.70(0.68–0.71)	0.83(0.81–0.85)	21.83(14.76–32.28)	0.9000±0.0140
P/I^2^	.0000/91.9%	.0000/64.7%	.0000/70.1%	-
SLE sample size				
n < 100	0.80(0.78–0.81)	0.77(0.74–0.80)	19.18(12.44–29.57)	0.8780±0.0174
P/I^2^	.0000/87.5%	.0000/63.4%	.0000/68.1%	-
n ≥ 100	0.65(0.63–0.67)	0.84(0.81–0.86)	13.25(7.97–22.03)	0.7565±0.0872
P/I^2^	.0005/77.3%	.0001/80.1%	.0005/77.2%	-

CI = confidence interval, DOR = diagnostic odds ratio, SROC = summary receiver operating characteristic curves value, AUC = area under the curve, SE = standard error, TmiRs = total mixed miRNAs, PBMC = peripheral blood mononuclear cell, SLE = systemic lupus erythematosus.

#### Publication bias

**Fig 6:**
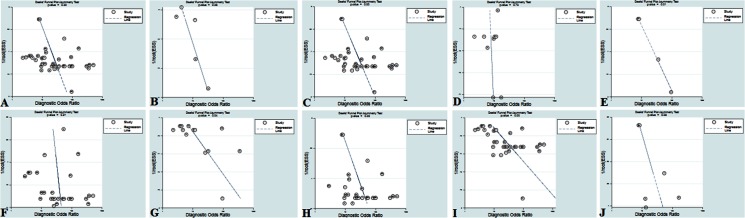
Funnel plot for the assessment of potential publication bias. The funnel graphs plot the square root of the effective sample size (1/ESS1/2) against the DOR. Each circle represents each study in the meta-analysis. Asymmetry of the circle distribution between regression lines indicates potential publication bias. (A) TmiRs pooled result, (B) miR-21 pooled result, (C) TmiRs-KO-21 pooled result, (D) PBMC pooled result, (E) serum pooled result, (F) plasma pooled result, (G) Caucasian pooled result, (H) Mongoloid pooled result, (I) SLE sample size (n < 100) pooled result, and (J) SLE sample size (n ≥ 100) pooled result. This funnel plot indicates no publication bias with a P value >.05. DOR = diagnostic odds ratio, ESS = effective sample size, miR = mircoRNA, TmiRs = total mixed miRNAs, TmiRs-KO-21 = total mixed miRNA knock out miR-21, SLE = systemic lupus erythematosus, PBMC = peripheral blood mononuclear cell.

We used funnel plots to evaluate the publication bias of the included studies. Each dot plots in these plots represented a study. The distance between each dot and the vertical line suggested bias in each study. The absence of any asymmetric distribution suggested that there was no publication bias. An asymmetric distribution indicated that publication bias existed. Deeks’ tests revealed the possibility of significant publication bias among the included evaluation pooled results of TmiRs (P < .001, 95%CI, 5.96 to 19.69) (Fig 6A), TmiRs-KO-21 (P = .000, 95%CI, 7.35 to 21.55) (Fig 6C), serum group (P = .006, 95%CI, 10.62 to 21.20) (Fig 6E), Caucasian group (P = .041, 95%CI, 2.44 to 95.45) (Fig 6G), Mongoloid group (P = .000, 95%CI, 8.56 to 23.44) (Fig 6H), and the SLE sample size (n < 100) group (P = .001, 95%CI, 26.40 to 97.65) (Fig 6I). Otherwise, there were no significant publication biases among the included studies of miR-21 (P = .077, 95%CI, –13.93 to 154.66) (Fig 6B), PBMC group (P = .732, 95%CI, –38.53 to 52.23) (Fig 6D), plasma group (P = .508, 95%CI, –18.80 to 37.01) (Fig 6F), and the SLE sample size (n ≥ 100) group (P = .081, 95%CI, -4.77 to 53.03) (Fig 6J) among the evaluation pooled results.

## Discussion

Since the introduction of miRNAs almost a decade ago, a series of publications have demonstrated the emerging role of miRNAs in the regulation of immune system development, maturation, and the pathological mechanisms of autoimmune disease, including SLE [43–47]. Furthermore, they can also be used in monitoring SLE disease severity function as novel potential targets for lupus treatment [47]. Meanwhile, many investigations had been carried out to assess whether miRNAs have sufficient capability to be used as biomarkers for SLE [48–50]. In this meta-analysis, we enrolled 17 studies and finally found that TmiRs achieved a combined AUC of 0.8797 with 71% pooled sensitivity, and 81% pooled specificity, which indicated that miRNAs had a moderate diagnostic accuracy as a diagnostic biomarker in discriminating SLE from healthy people. Furthermore, we examined single individual miRNAs among the total miRNA library and found that miR-21 was the most repeatedly used in individual studies recently. Based on this, we enrolled five studies on miR-21 and found a summary sensitivity of 0.68 (95%CI, 0.62 to 0.74) and summary specificity of 0.77 (95%CI, 0.69 to 0.84). In addition, the AUC value reached 0.8281±0.0608, which suggested that miR-21 had a moderate diagnostic accuracy in detecting SLE. Moreover, we also pooled the results of TmiRs after excluding the five studies on miR-21 for further confirmation. Although the AUC value rose slightly to 0.8895±0.0152 compared to that of TmiR, it still couldn’t deny the advantages of miR-21 in detecting SLE. To our knowledge, this is the first meta-analysis that focused on the accuracy of miR-21 in detecting SLE and confirmed the diagnostic accuracy of miRNAs as potential biomarkers for SLE.

miR-21 originated from chromosome 17q23.2 immediately to the vacuole membrane protein 1 gene and played an important role in regulating cell differentiation and proliferation [51–52]. Furthermore, miR-21 was considered as a promising biomarker of many diseases [53–55]. Also, previous studies suggested that miR-21 regulated aberrant responses of T cells of SLE [56–57], modulation of miR-21 expressions might restore the function of Treg cells and in turn reverse the clinical manifestations of SLE [19]. In lupus CD4+ T cells, the up-regulated miR-21 can suppress the expression of DNA methyltransferase 1 and enhance DNA hypomethylation [58]. However, the mechanism by which miR-21 regulates gene expression is still unclear and needs further research. However, miR-21 has been involved in several types of diseases, which would limit the diagnostic value of miR-21 in SLE with lower specificity compared to total miRNA. Give that, miR-21 is a key regulator in series of inflammation diseases, it still shows a significant role in differing related diseases such as autoimmune diseases and non-autoimmune diseases.

The results from our subgroup studies suggested that circulating miRNAs could be used as SLE diagnostic biomarkers in the Mongoloid group with relatively higher accuracy compared with the Caucasian group (the AUC value: 0.9000±0.0140, and 0.8028±0.0457, respectively). In addition, our meta-analysis showed that plasma-based specimens had a higher accuracy than PBMC or serum-based specimens (the AUC value: 0.9068±0.0151, 0.8044±0.0279, and 0.7073±0.0346, respectively). Besides, human miRNAs isolated from plasma are highly stable in boiling water and resistant to very high or low pH, prolonged room temperature incubation or repeated freeze-thawing [59]. Therefore, plasma is recommended as the clinical specimen for diagnostic detection.

Previous studies investigated the role of CRP, ERS, and neutrophil-to-lymphocyte ratio (NLR) in SLE patients associated with infection. Finally, they found that elevated CRP and NLR levels can be used as a predictor of active infection in SLE patients with a high specificity [60–62]. Our study aimed to gather evidence on how the diagnostic performance of miRNAs, especially miR-21, distinguished SLE with or without infection. Due to almost all of the included studies did not indicate whether SLE patients are associated with infectious diseases, we could not further analyze SLE patients who are infected and uninfected separately. It is worthy of note that the dysregulation of miRNAs in SLE could be the result of multiple factors [63]. Additionally, the ideal time for specimen acquisition was before treatment. Unfortunately, only two of the studies [17,31] involved in our meta-analysis indicated that blood samples were collected before treatment, while six studies [20,33,37,39,40,42] did not clarify when the specimens were obtained, five studies [18,19,21,36,41] stated that samples were collected during the period of treatments, and four articles [32,34,35,38] indicated that the time for specimen collection was after the patients had stopped treatments for a period of time. Thus, these differences in patient and blood collection time could cause inevitable bias to our results. Further well-designed studies with uniform criteria are needed to assess the diagnostic performance of miRNAs for SLE.

Additionally, the meta-analysis of six articles by Dai et al. found that the pooled sensitivity was 0.75, specificity was 0.72, and the AUC was 0.787 [22]. Our present meta-analysis showed that miRNAs had a relatively higher diagnostic accuracy. More importantly, the advantages of our meta-analysis are as follows: first, this study was much larger, with more than twice as many studies as the earlier meta-analysis. Second, we performed a meta-analysis of five reports to verify the diagnostic accuracy of miR-21 as potential biomarkers for SLE, which was a key miRNA among the total miRNAs. Third, we carried out the meta-regression analysis detect where the potential factor for heterogeneity origin from and found the type of samples might be the sources of heterogeneity. Furthermore, the subgroup analyses detected that miRNAs in plasma had the highest diagnostic accuracy.

## Study limitations

As some pooled results showed large heterogeneities, several limitations of this meta-analysis need to be addressed. First, our meta-analysis included 39 miRNA markers, with only six miRNA markers that were repeatedly identified in two or three of the included publications except for miR-21, so that we did not conduct a meta-analysis on the same individual miRNA markers across publications except for miR-21. Second, due to the lack of conventional methodologies for an accurate absolute quantification of miRNAs, which limits the cross-comparison between studies performed by different laboratories, might produce unconvincing results for the included studies. Third, no included articles combine miRNAs with other laboratory tests, such as complement and antibodies to identify the diagnostic accuracy of SLE, which could work as a better method for detection. Besides, the times of sample obtained were varied among included studies, which might generated bias and decrease the strength in interpreting the results.

## Conclusions

In conclusion, despite these limitations, our meta-analysis demonstrated that circulating miRNAs might be potential novel biomarkers for detecting SLE, especially miR-21. Moreover, plasma is recommended as the clinical specimen for diagnostic detection. Therefore, more well-designed researches need to be done to launch the application of miRNAs as biomarkers for SLE detection in the clinic. Furthermore, a combination of miRNAs and other laboratory tests needs to be worked as a better method for detection.

## Supporting information

S1 Table PRISMA checklist. (DOC)

S1 Appendix Search strategies for EMBASE, the Cochrane Central Register of Controlled Trials, and China National Knowledge Infrastructure. (DOCX)

S1 Fig Performance of TmiRs-KO-21 detection for the diagnosis of SLE. (A) Pooled sensitivity. (B) Pooled specificity. (C) Overall DOR. (D) The SROC curves for all data sets. The point estimates from each study are shown as solid squares. The pooled estimates are shown as a solid diamond. Effect sizes were pooled by random-effects models. Each square in the SROC curve represents one study. Sample size is indicated by the size of the square. Error bars represent 95% CIs. CI, confidence interval; miR, mircoRNA; SROC; summary receiver operating characteristic curves value; OR, odds ratio.
(TIF)

S2 Fig Performance of PBMC detection for the diagnosis of SLE. (A) Pooled sensitivity. (B) Pooled specificity. (C) Overall DOR. (D) The SROC curves for all data sets. The point estimates from each study are shown as solid squares. The pooled estimates are shown as a solid diamond. Effect sizes were pooled by random-effects models. Each square in the SROC curve represents one study. Sample size is indicated by the size of the square. Error bars represent 95% CIs. CI, confidence interval; miR, mircoRNA; SROC; summary receiver operating characteristic curves value; OR, odds ratio.
(TIF)

S3 Fig Performance of serum detection for the diagnosis of SLE. (A) Pooled sensitivity. (B) Pooled specificity. (C) Overall DOR. (D) The SROC curves for all data sets. The point estimates from each study are shown as solid squares. The pooled estimates are shown as a solid diamond. Effect sizes were pooled by random-effects models. Each square in the SROC curve represents one study. Sample size is indicated by the size of the square. Error bars represent 95% CIs. CI, confidence interval; miR, mircoRNA; SROC; summary receiver operating characteristic curves value; OR, odds ratio.
(TIF)

S4 Fig Performance of plasma detection for the diagnosis of SLE. (A) Pooled sensitivity. (B) Pooled specificity. (C) Overall DOR. (D) The SROC curves for all data sets. The point estimates from each study are shown as solid squares. The pooled estimates are shown as a solid diamond. Effect sizes were pooled by random-effects models. Each square in the SROC curve represents one study. Sample size is indicated by the size of the square. Error bars represent 95% CIs. CI, confidence interval; miR, mircoRNA; SROC; summary receiver operating characteristic curves value; OR, odds ratio.
(TIF)

S5 Fig Performance of Caucasian detection for the diagnosis of SLE. (A) Pooled sensitivity. (B) Pooled specificity. (C) Overall DOR. (D) The SROC curves for all data sets. The point estimates from each study are shown as solid squares. The pooled estimates are shown as a solid diamond. Effect sizes were pooled by random-effects models. Each square in the SROC curve represents one study. Sample size is indicated by the size of the square. Error bars represent 95% CIs. CI, confidence interval; miR, mircoRNA; SROC; summary receiver operating characteristic curves value; OR, odds ratio.
(TIF)

S6 Fig Performance of Mongoloid detection for the diagnosis of SLE. (A) Pooled sensitivity. (B) Pooled specificity. (C) Overall DOR. (D) The SROC curves for all data sets. The point estimates from each study are shown as solid squares. The pooled estimates are shown as a solid diamond. Effect sizes were pooled by random-effects models. Each square in the SROC curve represents one study. Sample size is indicated by the size of the square. Error bars represent 95% CIs. CI, confidence interval; miR, mircoRNA; SROC; summary receiver operating characteristic curves value; OR, odds ratio.
(TIF)

S7 Fig Performance of SLE sample size (n < 100) detection for the diagnosis of SLE. (A) Pooled sensitivity. (B) Pooled specificity. (C) Overall DOR. (D) The SROC curves for all data sets. The point estimates from each study are shown as solid squares. The pooled estimates are shown as a solid diamond. Effect sizes were pooled by random-effects models. Each square in the SROC curve represents one study. Sample size is indicated by the size of the square. Error bars represent 95% CIs. CI, confidence interval; miR, mircoRNA; SROC; summary receiver operating characteristic curves value; OR, odds ratio.
(TIF)

S8 Fig Performance of SLE sample size (n ≥ 100) detection for the diagnosis of SLE. (A) Pooled sensitivity. (B) Pooled specificity. (C) Overall DOR. (D) The SROC curves for all data sets. The point estimates from each study are shown as solid squares. The pooled estimates are shown as a solid diamond. Effect sizes were pooled by random-effects models. Each square in the SROC curve represents one study. Sample size is indicated by the size of the square. Error bars represent 95% CIs. CI, confidence interval; miR, mircoRNA; SROC; summary receiver operating characteristic curves value; OR, odds ratio.
(TIF)
