# Supplementary material for: Diagnostic significance of circulating miRNAs in systemic lupus erythematosus
Source: PLoS One. 2019 Jun 4;14(6):e0217523. doi: 10.1371/journal.pone.0217523 (PMC6548426; doi:10.1371/journal.pone.0217523)
Supplement: S1 Table — (DOC) [file pone.0217523.s001.doc]

| **Section/topic** | **#** | **Checklist item** | **Reported on page #** |
| --- | --- | --- | --- |
| **TITLE** | | |  |
| Title | 1 | Diagnostic significance of circulating miRNAs in systemic lupus erythematosus (a meta-analysis) | 1 |
| ABSTRACT | | |  |
| Structured summary | 2 | Background  In recent years, many studies focused on the association between the microRNAs (miRNAs) and the risk of systemic lupus erythematosus (SLE), especially miRNA-21 (miR-21). We aimed to investigate the role of circulating miRNAs, especially the miR-21, as a biomarker in detecting SLE.  Methods  We searched PubMed, EMBASE, the Cochrane Central Register of Controlled Trials, and China National Knowledge Infrastructure through Mar 3th, 2019. We performed this meta-analysis in a fixed/random-effect model using Meta-disc 1.4 and STATA 15.1. A total of 17 relevant studies were eligible to analyze pooled accuracy. The overall performance of total mixed miRNAs (TmiRs) detection was: pooled sensitivity, 0.71 (95% confidence interval [CI], 0.69 to 0.72); pooled specificity, 0.81 (95%CI, 0.79 to 0.83); and area under the summary receiver operating characteristic curves value (SROC), 0.8797. The miR-21 detection was: pooled sensitivity, 0.68 (95%CI, 0.62 to 0.74); pooled specificity, 0.77 (95%CI, 0.69 to 0.84); and SROC, 0.8281. The meta-regression analysis showed that the type of sample was the sources of heterogeneity. The subgroup analysis suggested that detection in plasma group had the largest AUC of SROC in all the subgroups: pooled sensitivity, 0.8 (95%CI, 0.78 to 0.82); pooled specificity, 0.83 (95%CI, 0.8 to 0.86); and SROC, 0.9068.  Conclusions  Our meta-analysis demonstrated that circulating miRNAs might be potential novel biomarkers for detecting SLE, especially miR-21. Moreover, plasma is recommended as the clinical specimen for diagnostic detection. | 1-2 |
| INTRODUCTION | | |  |
| Rationale | 3 | Systemic lupus erythematosus (SLE) is a complex, chronic, potentially fatal, multisystem autoimmune disease, which predominantly affects women between puberty and menopause. The major pathogenetic mechanisms of SLE include an inappropriate immune response to the nucleic acid containing cellular particles, which is caused by an autoimmune reaction of the innate and adaptive immune systems, leading to damage structures of the skin, joints, kidney and central nervous system. Mortality in SLE patients has improved over the past 30 years but remains considerably higher than in people from the same geographical area without SLE. Delay in diagnosis is associated with increased damage to vital organs. The diagnosis of SLE is very challenging because there are no generally accepted diagnostic criteria. Therefore, it is important to find novel and reliable circulating biomarkers for the early diagnosis of SLE.  MicroRNAs (miRNAs) are a class of endogenously small noncoding RNAs with approximately 18-25 nucleotides in length, and may inhibit protein translation or degrading the polypeptides via binding to the untranslated regions of mRNA. Previous studies have found that circulating miRNAs can be considered as potential biomarkers for detecting kinds of diseases, including autoimmune diseases. In 2011, Stagaki et al. suggested that miRNA-21 (miR-21) was a potential biomarker for SLE. Since then, a series of studies have also verified the role of miR-21 in SLE. In addition, these studies have found other miRNAs (miR-155, -181a, -196a, -31, and -148a) dysregulated in SLE. Dai et al carried out a meta-analysis to verify the diagnostic accuracy of miRNAs as potential biomarkers for SLE. However, many new studies focused on the association between the miRNAs and the risk of SLE were reported in recent years, especially the relationship between miR-21and SLE. | 2-3 |
| Objectives | 4 | We collected all published case-control studies to gather evidence on how the diagnostic performance of miRNAs, especially miR-21, distinguished SLE. | 3 |
| METHODS | | |  |
| Protocol and registration | 5 | This analysis was performed by a predetermined protocol following the recommendations of Deeks. The data collection and reporting accorded with the Preferred Reporting Items for Systematic Reviews and Meta-Analyses (PRISMA) Statement. The ethical approval was not necessary due to it is systematic literature research. | 3 |
| Eligibility criteria | 6 | Criteria for inclusion: (1) all patients of SLE were confirmed by SLE diagnosis criteria; (2) randomized controlled or non- randomized controlled, clinical trials, cohort studies evaluating the expression of miRNAs; (3) contained the data of true positive, false positive, false negative, and true negative; or the data of the receiver operating characteristic (ROC) curve, and essential sample size; (4) all studies had healthy controls; (5) real-time PCR (RT-PCR), qRT-PCR, microarray, and miRNA sequence were acceptable methods to evaluate the expressions of miRNAs; (6) full text published in English.  Criteria for Exclusion: (1) conferences articles, reviews, letters, or case reports without controls; (2) no available data to construct a 2×2 table; (3) duplicated reports. | 4 |
| Information sources | 7 | We searched multiple databases including PubMed, EMBASE, the Cochrane Central Register of Controlled Trials, and China National Knowledge Infrastructure through Mar 3th, 2019 to identify relevant studies. | 3 |
| Search | 8 | Keyword search terms were (‘systemic lupus erythematosus’ OR ‘lupus erythematosus’ OR ‘lupus nephritis’) AND (‘MicroRNAs’ OR ‘MicroRNA’ OR ‘miRNAs’ OR ‘miRNA’). PubMed database was searched as follows: (Lupus Erythematosus, Systemic[MeSH Terms] OR systemic lupus erythematosus OR lupus erythematosus OR lupus nephritis) AND (MicroRNAs[MeSH Terms] OR MicroRNA OR miRNAs OR miRNA). | 3 |
| Study selection | 9 | Reports were preliminarily screened by title and abstract and when initially selected by the systematic search. Potentially relevant studies were then retrieved by full manuscripts and assessed for compliance with inclusion and exclusion criteria. | 4 |
| Data collection process | 10 | Two investigators (Xiaolan Zheng, Yi Zhang) screened and assessed the eligibility of reports at the title and/or abstract level independently according to the inclusion and exclusion criteria., and a third reviewer (Yifei Li) determining the divergences according to inclusion or exclusion criteria, and the quality of reports; studies that met all the inclusion criteria were selected for further analysis. According to the 14-item Quality Assessment of Diagnostic Accuracy Studies (QUADAS) list, the quality assessment of all enrolled studies was independently conducted by two investigators (Xiaolan Zheng, Peng Yue), and any disagreement was settled by discussion. As a well-conducted study might score poorly once related parts were missing among the methods and results so that all the assessments were only reported in descriptive forms. We used Photoshop CS6 (Adobe Systems Software Ireland Ltd) to extract data from the figures. By Photoshop CS6, we were able to set axes and identify the abscissa and ordinate of each point. Finally, two investigators (Xiaolan Zheng, Lei Liu) extracted the date which can calculate true positive, false positive, false negative, and true negative, such as sensitivity, specificity, and essential sample size. | 4-5 |
| Data items | 11 | the data of true positive, false positive, false negative, and true negative; or the data of the receiver operating characteristic (ROC) curve, and essential sample size | 4 |
| Risk of bias in individual studies | 12 | We used Stata statistical software (STATA, version 15.1) to obtain a quantitative analysis of all the publication bias according to funnel plots and the Deek’s test. An asymmetric distribution of data points in the funnel plot with a quantified result of P<.05 indicated the presence of potential publication bias | 5 |
| Summary measures | 13 | The following indicators of different types of miRNAs were measured: sensitivity, specificity, diagnostic odds ratio (DOR), and area under the summary receiver operating characteristic curves value (SROC). | 5 |
| Synthesis of results | 14 | Sensitivity, specificity, diagnostic odds ratio (DOR), and area under the summary receiver operating characteristic curves value (SROC). | 5 |

Page 1 of 2

| **Section/topic** | **#** | **Checklist item** | **Reported on page #** |
| --- | --- | --- | --- |
| Risk of bias across studies | 15 | We used Stata statistical software (STATA, version 15.1) to obtain a quantitative analysis of all the publication bias according to funnel plots and the Deek’s test. An asymmetric distribution of data points in the funnel plot with a quantified result of P<.05 indicated the presence of potential publication bias | 5 |
| Additional analyses | 16 | We carried out the meta-regression analysis using STATA 15.1 to detect where the potential factor for heterogeneity origin from. Sensitivity analysis was conducted for every study to determine the influence of individual trials on the results, using STATA 15.1 for meta-analysis fixed/random-effects estimates. Meta-Disc 1.4 was used to detect threshold effects in studies and conduct subgroup analysis. | 5-6 |
| RESULTS | | |  |
| Study selection | 17 | Initially, 1301 potentially relevant papers were retrieved by the search method aforementioned, of which 33 articles were considered to be interested after reading titles and abstracts. However, five articles were excluded by reading their complete articles due to article types, seven studies lacked available data to construct a 2×2 table, and four articles lacked a comparison between SLE patients and healthy controls. Finally, 17 studies were included in the meta-analysis. | 6 |
| Study characteristics | 18 | Except the study of Zununi Vahed et al. is a cross-sectional trial, all others are prospective trials. Among them, 41 individual diagnostic tests were extracted for total mixed miRNA (TmiR) evaluation; five individual diagnostic tests were extracted for miR-21 evaluation, 36 individual diagnostic tests were enrolled for total mixed miRNA knock out miR-21 (TmiRs-KO-21) evaluation. Additionally, the sample types of nine studies were plasma, five studies were peripheral blood mononuclear cells (PBMC), and the remaining three studies were serum. Moreover, there are ten reports from China, in which ethnicity can be defined as Mongoloid; and seven studies from other different countries (two from Iran, one from Bulgaria, three from Egypt, and one from Colombia), in which ethnicity can be defined as Caucasian. Besides, the SLE sample size of 13 studies were much smaller (n < 100) compared the remaining four (n ≥ 100). | 6 |
| Risk of bias within studies | 19 | We used funnel plots to evaluate the publication bias of the included studies. Each dot plots in these plots represented a study. The distance between each dot and the vertical line suggested bias in each study. The absence of any asymmetric distribution suggested that there was no publication bias. An asymmetric distribution indicated that publication bias existed. Deeks’ tests revealed the possibility of significant publication bias among the included evaluation pooled results of TmiRs (P＜.001, 95%CI, 5.96 to 19.69), TmiRs-KO-21 (P=.000, 95%CI, 7.35 to 21.55), serum group (P=.006, 95%CI, 10.62 to 21.20), Caucasian group (P=.041, 95%CI, 2.44 to 95.45), Mongoloid group (P=.000, 95%CI, 8.56 to 23.44), and the SLE sample size (n < 100) group (P=.001, 95%CI, 26.40 to 97.65). Otherwise, there were no significant publication biases among the included studies of miR-21 (P=.077, 95%CI, –13.93 to 154.66), PBMC group (P=.732, 95%CI, –38.53 to 52.23), plasma group (P=.508, 95%CI, –18.80 to 37.01), and the SLE sample size (n ≥ 100) group (P=.081, 95%CI, -4.77 to 53.03) among the evaluation pooled results. | 9 |
| Results of individual studies | 20 | A total of 17 relevant studies were eligible to analyze pooled accuracy. The overall performance of total mixed miRNAs (TmiRs) detection was: pooled sensitivity, 0.71 (95% confidence interval [CI], 0.69 to 0.72); pooled specificity, 0.81 (95%CI, 0.79 to 0.83); and area under the summary receiver operating characteristic curves value (SROC), 0.8797. The miR-21 detection was: pooled sensitivity, 0.68 (95%CI, 0.62 to 0.74); pooled specificity, 0.77 (95%CI, 0.69 to 0.84); and SROC, 0.8281. The meta-regression analysis showed that the type of samples was the sources of heterogeneity. The subgroup analysis suggested that detection in plasma group had the largest AUC of SROC in all the subgroups: pooled sensitivity, 0.8 (95%CI, 0.78 to 0.82); pooled specificity, 0.83 (95%CI, 0.8 to 0.86); and SROC, 0.9068. | 7-9 |
| Synthesis of results | 21 | The overall performance of total mixed miRNAs (TmiRs) detection was: pooled sensitivity, 0.71 (95% confidence interval [CI], 0.69 to 0.72); pooled specificity, 0.81 (95%CI, 0.79 to 0.83); and area under the summary receiver operating characteristic curves value (SROC), 0.8797. The miR-21 detection was: pooled sensitivity, 0.68 (95%CI, 0.62 to 0.74); pooled specificity, 0.77 (95%CI, 0.69 to 0.84); and SROC, 0.8281. The meta-regression analysis showed that the type of samples was the sources of heterogeneity. The subgroup analysis suggested that detection in plasma group had the largest AUC of SROC in all the subgroups: pooled sensitivity, 0.8 (95%CI, 0.78 to 0.82); pooled specificity, 0.83 (95%CI, 0.8 to 0.86); and SROC, 0.9068. | 7-9 |
| Risk of bias across studies | 22 | We used funnel plots to evaluate the publication bias of the included studies. Each dot plots in these plots represented a study. The distance between each dot and the vertical line suggested bias in each study. The absence of any asymmetric distribution suggested that there was no publication bias. An asymmetric distribution indicated that publication bias existed. Deeks’ tests revealed the possibility of significant publication bias among the included evaluation pooled results of TmiRs (P＜.001, 95%CI, 5.96 to 19.69), TmiRs-KO-21 (P=.000, 95%CI, 7.35 to 21.55), serum group (P=.006, 95%CI, 10.62 to 21.20), Caucasian group (P=.041, 95%CI, 2.44 to 95.45), Mongoloid group (P=.000, 95%CI, 8.56 to 23.44), and the SLE sample size (n < 100) group (P=.001, 95%CI, 26.40 to 97.65). Otherwise, there were no significant publication biases among the included studies of miR-21 (P=.077, 95%CI, –13.93 to 154.66), PBMC group (P=.732, 95%CI, –38.53 to 52.23), plasma group (P=.508, 95%CI, –18.80 to 37.01), and the SLE sample size (n ≥ 100) group (P=.081, 95%CI, -4.77 to 53.03) among the evaluation pooled results. | 9 |
| Additional analysis | 23 | The meta-regression also found the SLE sample sizes is not a dramatic impact factor, P=.77, t=-0.30, 95%CI (0.29, 2.52). Therefore, the type of samples might be responsible for the existing heterogeneities. The results showed that the Spearman correlation coefficient was 0.178 and P=.264, meaning no threshold effect related to heterogeneity existed. Among the all enrolled studies, there were three different types of sample collection protocols (plasma, serum, and PBMC), two ethnicities (Caucasian and Mongoloid), and two SLE sample sizes (n < 100 and n ≥ 100). And then, we conducted three subgroups analysis according to the type of samples, ethnicity, and the SLE sample size. The subgroup analysis suggested that the plasma group had the largest AUC of SROC in all the subgroups: pooled sensitivity, 0.8 (95%CI, 0.78 to 0.82); pooled specificity, 0.83 (95%CI, 0.8 to 0.86); and SROC, 0.9068. | 7-9 |
| **DISCUSSION** | | |  |
| Summary of evidence | 24 | The overall performance of total mixed miRNAs (TmiRs) detection was: pooled sensitivity, 0.71 (95% confidence interval [CI], 0.69 to 0.72); pooled specificity, 0.81 (95%CI, 0.79 to 0.83); and area under the summary receiver operating characteristic curves value (SROC), 0.8797. The miR-21 detection was: pooled sensitivity, 0.68 (95%CI, 0.62 to 0.74); pooled specificity, 0.77 (95%CI, 0.69 to 0.84); and SROC, 0.8281. | 9-10 |
| Limitations | 25 | As some pooled results showed large heterogeneities, several limitations of this meta-analysis need to be addressed. First, our meta-analysis included 39 miRNA markers, with only six miRNA markers that were repeatedly identified in two or three of the included publications except for miR-21, so that we did not conduct a meta-analysis on the same individual miRNA markers across publications except for miR-21. Second, due to the lack of conventional methodologies for an accurate absolute quantification of miRNAs, which limits the cross-comparison between studies performed by different laboratories, might produce unconvincing results for the included studies. Third, no included articles combine miRNAs with other laboratory tests, such as complement and antibodies to identify the diagnostic accuracy of SLE, which could work as a better method for detection | 10-11 |
| Conclusions | 26 | In conclusion, despite these limitations, our meta-analysis demonstrated that circulating miRNAs might be potential novel biomarkers for detecting SLE, especially miR-21. Moreover, plasma is recommended as the clinical specimen for diagnostic detection. Therefore, more research needs to be done to launch the application of miRNAs as biomarkers for SLE detection in the clinic. Furthermore, a combination of miRNAs and other laboratory tests needs to be worked as a better method for detection. | 11 |
| **FUNDING** | | |  |
| Funding | 27 | This work was supported by grants from the National Natural Science Foundation of China (No. 81700360, 81741025, 81570369 and 81571515) and the Technologic Program from Sichuan Province Government Foundation, China (No. 2018JY0603). |  |

*From:*  Moher D, Liberati A, Tetzlaff J, Altman DG, The PRISMA Group (2009). Preferred Reporting Items for Systematic Reviews and Meta-Analyses: The PRISMA Statement. PLoS Med 6(7): e1000097. doi:10.1371/journal.pmed1000097

For more information, visit: **www.prisma-statement.org**.

Page 2 of 2
