# Supplementary figures and images for: Diagnostic significance of circulating miRNAs in systemic lupus erythematosus
Source: PLoS One. 2019 Jun 4;14(6):e0217523. doi: 10.1371/journal.pone.0217523 (PMC6548426; doi:10.1371/journal.pone.0217523)

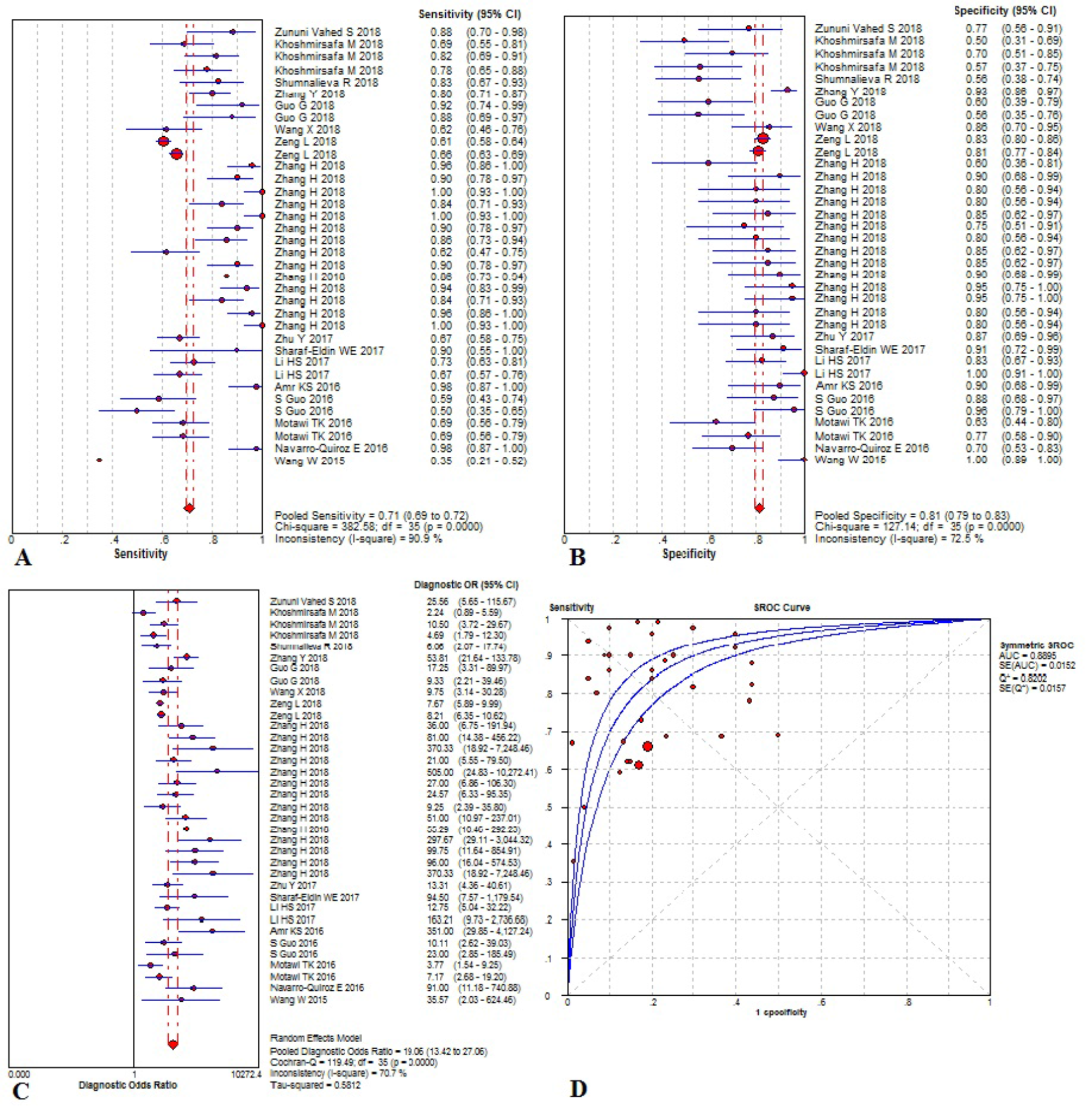

Supplement: S1 Fig — (A) Pooled sensitivity. (B) Pooled specificity. (C) Overall DOR. (D) The SROC curves for all data sets. The point estimates from each study are shown as solid squares. The pooled estimates are shown as a solid diamond. Effect sizes were pooled by random-effects models. Each square in the SROC curve represents one study. Sample size is indicated by the size of the square. Error bars represent 95% CIs. CI, confidence interval; miR, mircoRNA; SROC; summary receiver operating characteristic curves value; OR, odds ratio. (TIF) [file pone.0217523.s003.tif]

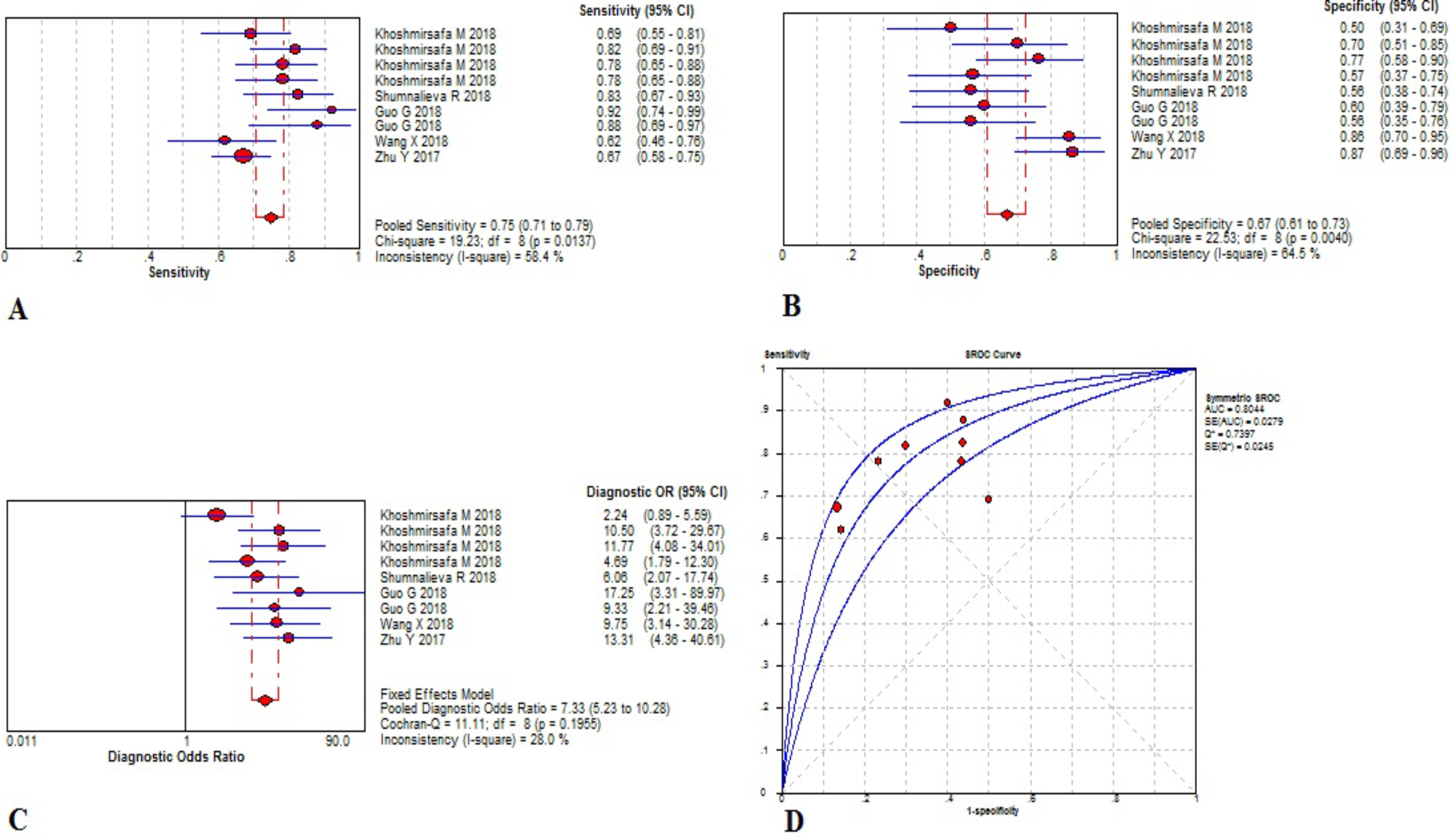

Supplement: S2 Fig — (A) Pooled sensitivity. (B) Pooled specificity. (C) Overall DOR. (D) The SROC curves for all data sets. The point estimates from each study are shown as solid squares. The pooled estimates are shown as a solid diamond. Effect sizes were pooled by random-effects models. Each square in the SROC curve represents one study. Sample size is indicated by the size of the square. Error bars represent 95% CIs. CI, confidence interval; miR, mircoRNA; SROC; summary receiver operating characteristic curves value; OR, odds ratio. (TIF) [file pone.0217523.s004.tif]

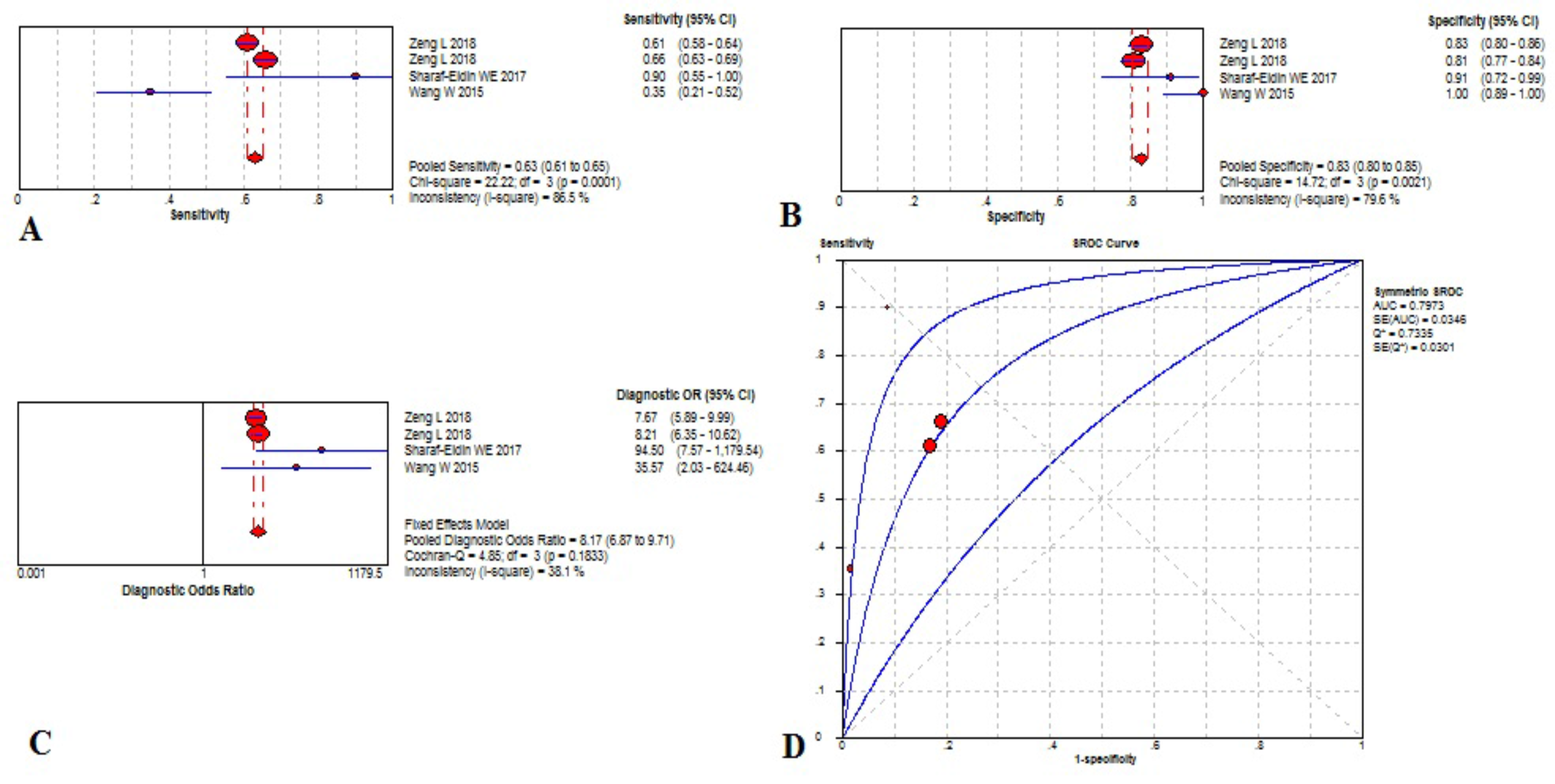

Supplement: S3 Fig — (A) Pooled sensitivity. (B) Pooled specificity. (C) Overall DOR. (D) The SROC curves for all data sets. The point estimates from each study are shown as solid squares. The pooled estimates are shown as a solid diamond. Effect sizes were pooled by random-effects models. Each square in the SROC curve represents one study. Sample size is indicated by the size of the square. Error bars represent 95% CIs. CI, confidence interval; miR, mircoRNA; SROC; summary receiver operating characteristic curves value; OR, odds ratio. (TIF) [file pone.0217523.s005.tif]

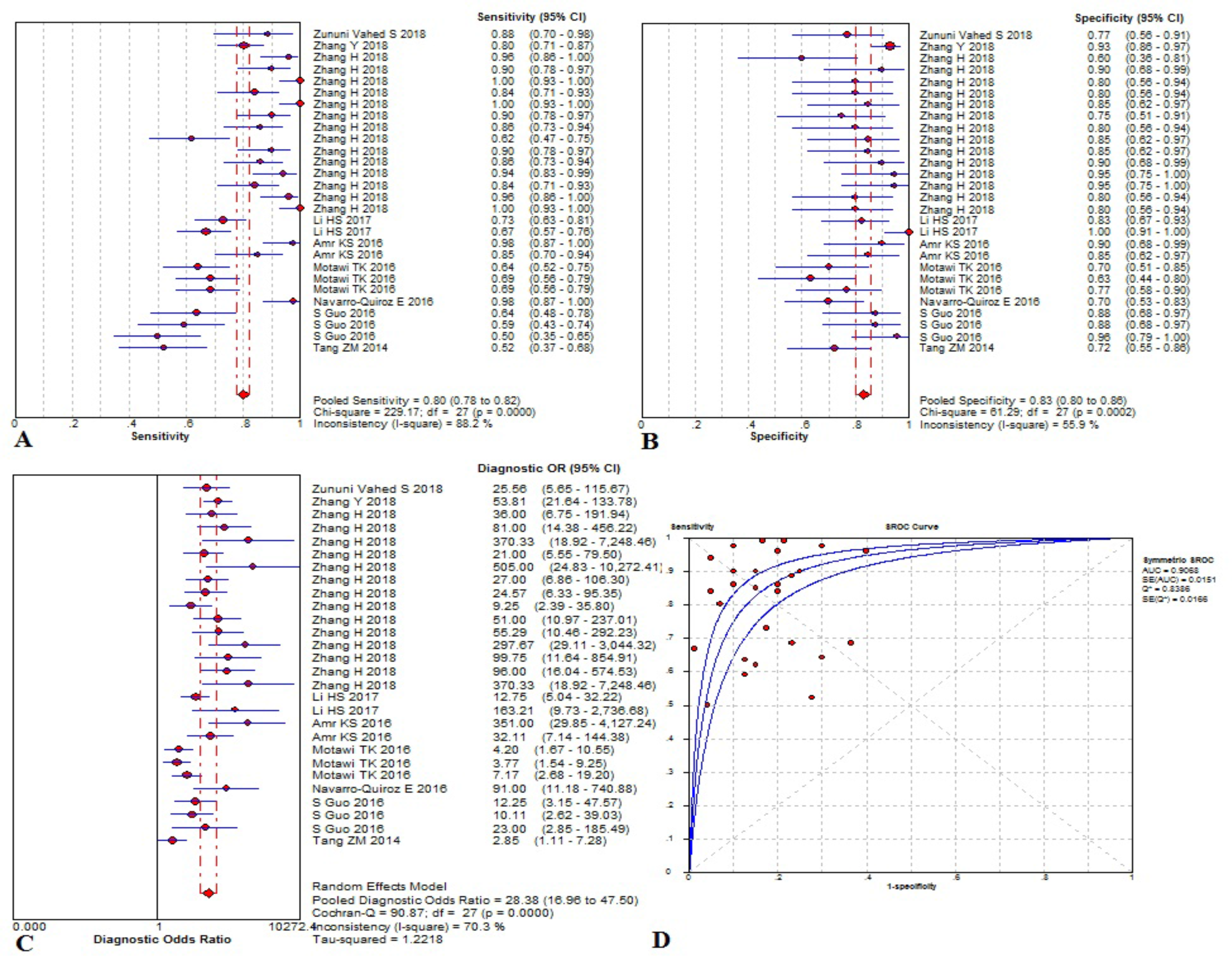

Supplement: S4 Fig — (A) Pooled sensitivity. (B) Pooled specificity. (C) Overall DOR. (D) The SROC curves for all data sets. The point estimates from each study are shown as solid squares. The pooled estimates are shown as a solid diamond. Effect sizes were pooled by random-effects models. Each square in the SROC curve represents one study. Sample size is indicated by the size of the square. Error bars represent 95% CIs. CI, confidence interval; miR, mircoRNA; SROC; summary receiver operating characteristic curves value; OR, odds ratio. (TIF) [file pone.0217523.s006.tif]

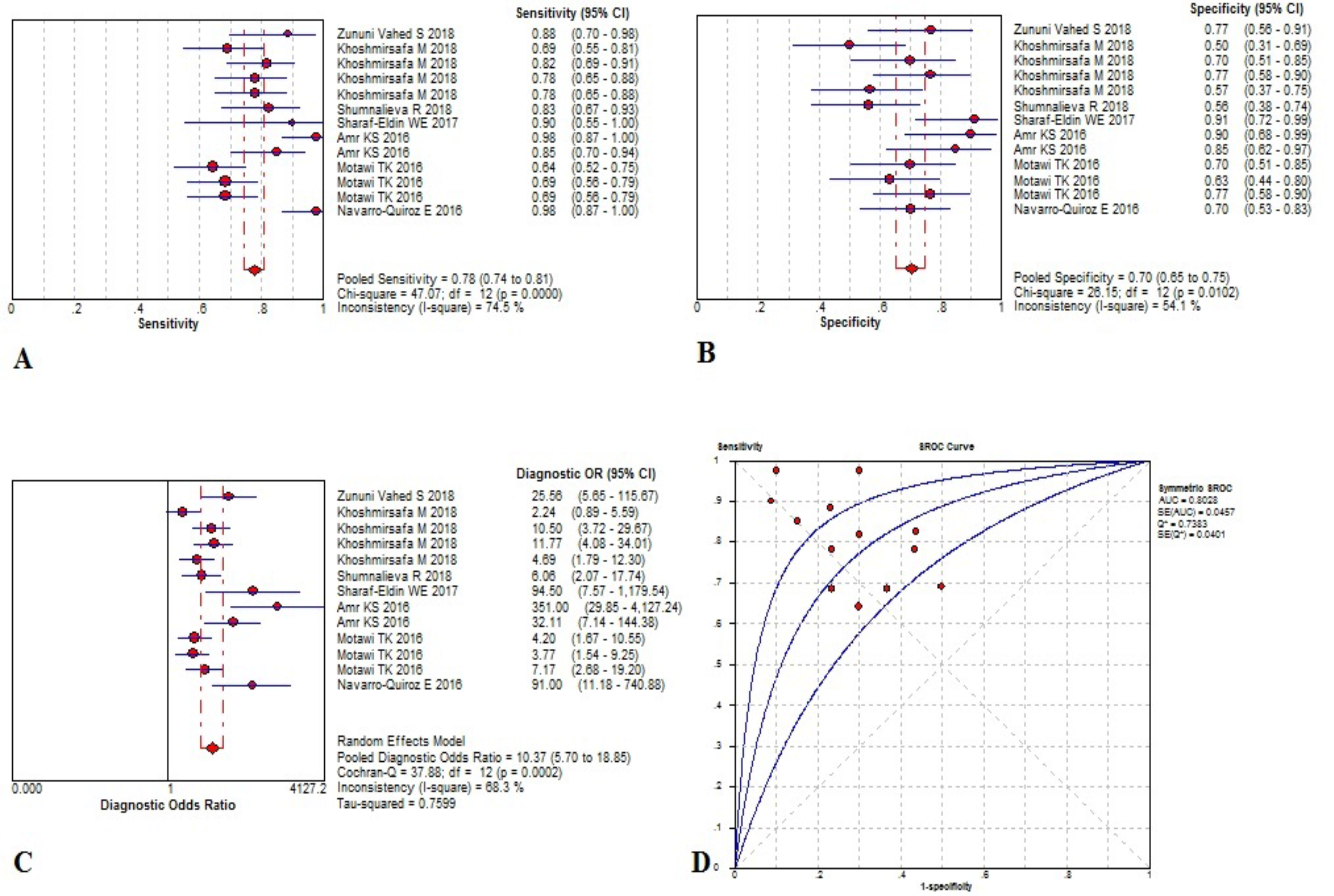

Supplement: S5 Fig — (A) Pooled sensitivity. (B) Pooled specificity. (C) Overall DOR. (D) The SROC curves for all data sets. The point estimates from each study are shown as solid squares. The pooled estimates are shown as a solid diamond. Effect sizes were pooled by random-effects models. Each square in the SROC curve represents one study. Sample size is indicated by the size of the square. Error bars represent 95% CIs. CI, confidence interval; miR, mircoRNA; SROC; summary receiver operating characteristic curves value; OR, odds ratio. (TIF) [file pone.0217523.s007.tif]

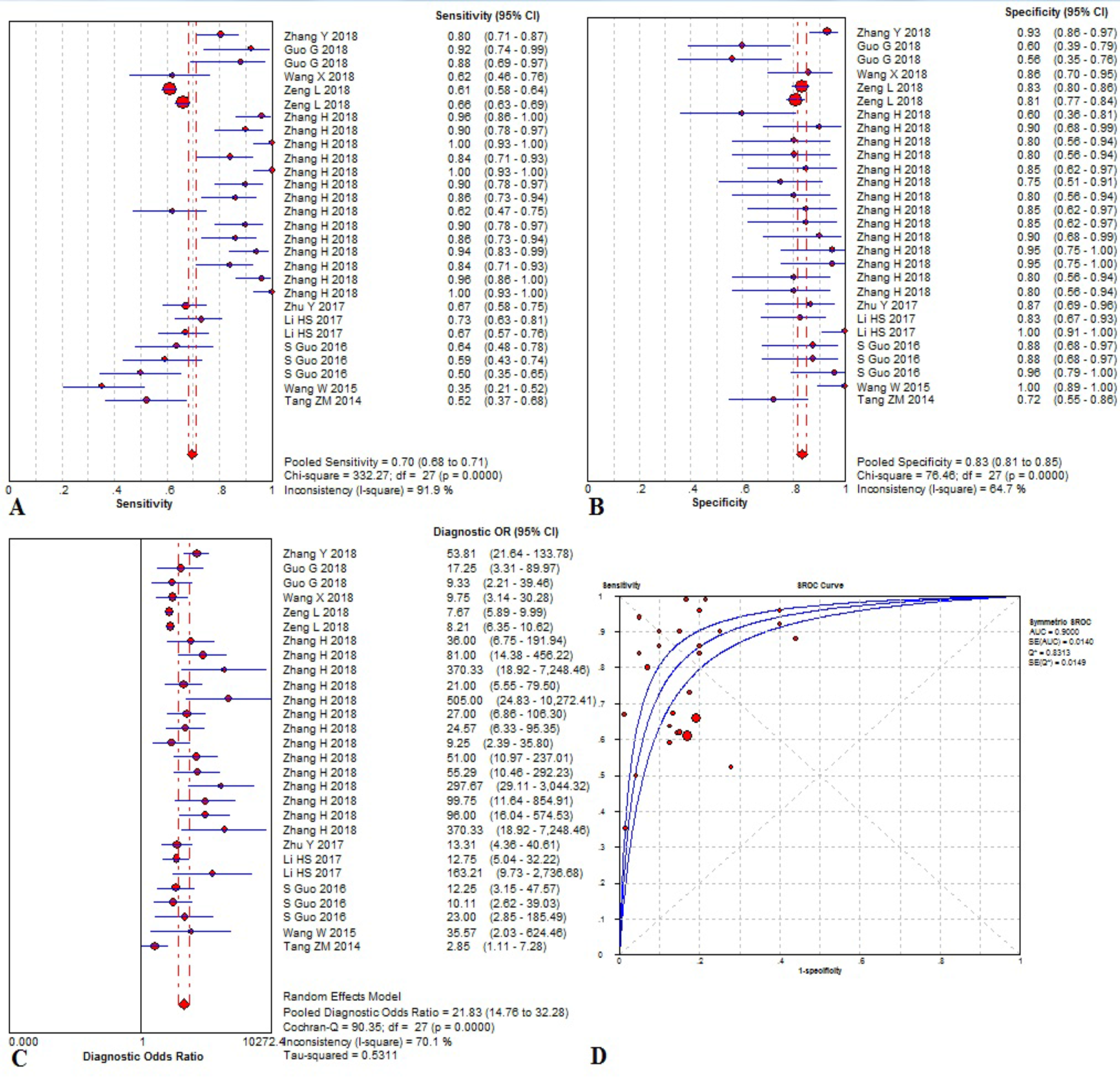

Supplement: S6 Fig — (A) Pooled sensitivity. (B) Pooled specificity. (C) Overall DOR. (D) The SROC curves for all data sets. The point estimates from each study are shown as solid squares. The pooled estimates are shown as a solid diamond. Effect sizes were pooled by random-effects models. Each square in the SROC curve represents one study. Sample size is indicated by the size of the square. Error bars represent 95% CIs. CI, confidence interval; miR, mircoRNA; SROC; summary receiver operating characteristic curves value; OR, odds ratio. (TIF) [file pone.0217523.s008.tif]

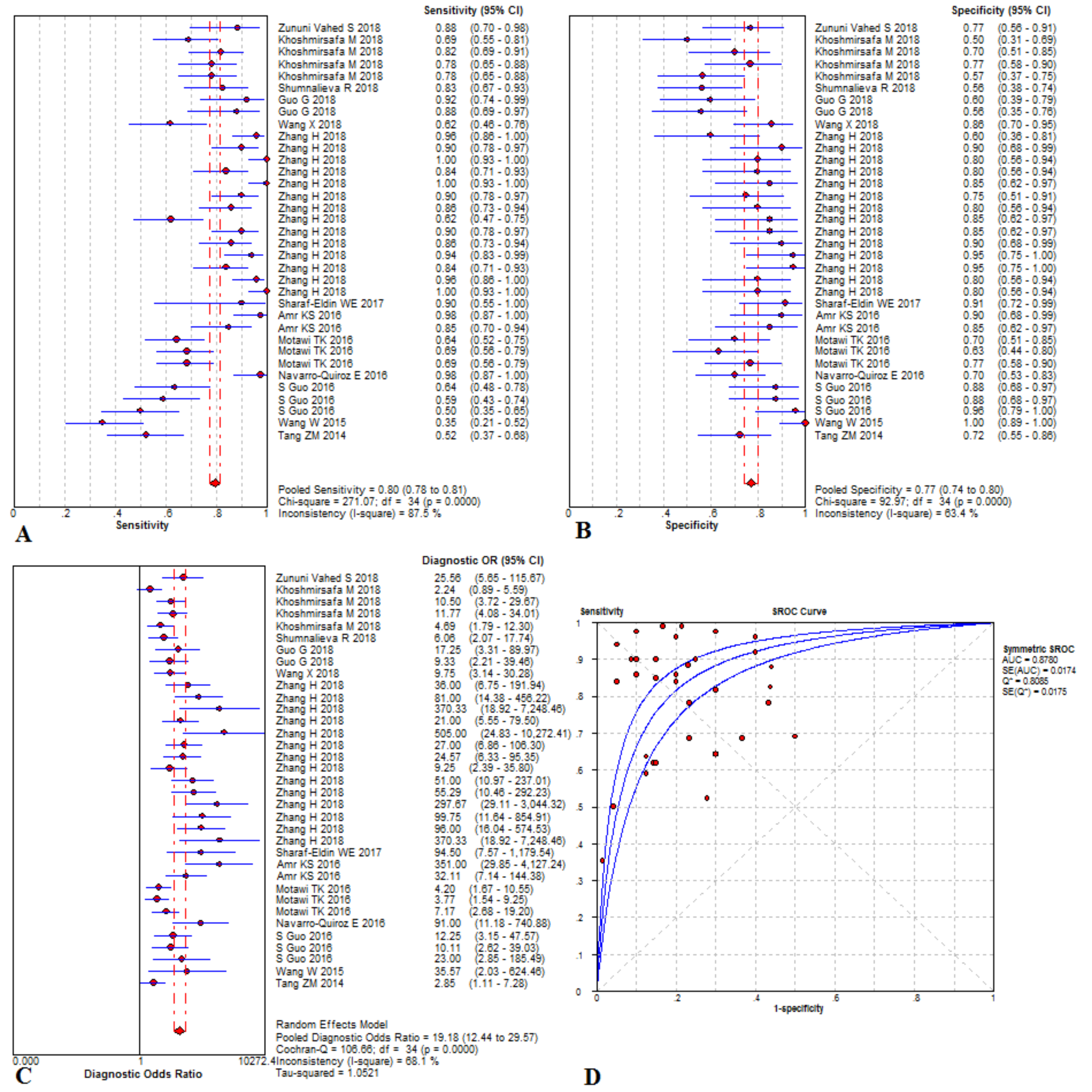

Supplement: S7 Fig — (A) Pooled sensitivity. (B) Pooled specificity. (C) Overall DOR. (D) The SROC curves for all data sets. The point estimates from each study are shown as solid squares. The pooled estimates are shown as a solid diamond. Effect sizes were pooled by random-effects models. Each square in the SROC curve represents one study. Sample size is indicated by the size of the square. Error bars represent 95% CIs. CI, confidence interval; miR, mircoRNA; SROC; summary receiver operating characteristic curves value; OR, odds ratio. (TIF) [file pone.0217523.s009.tif]

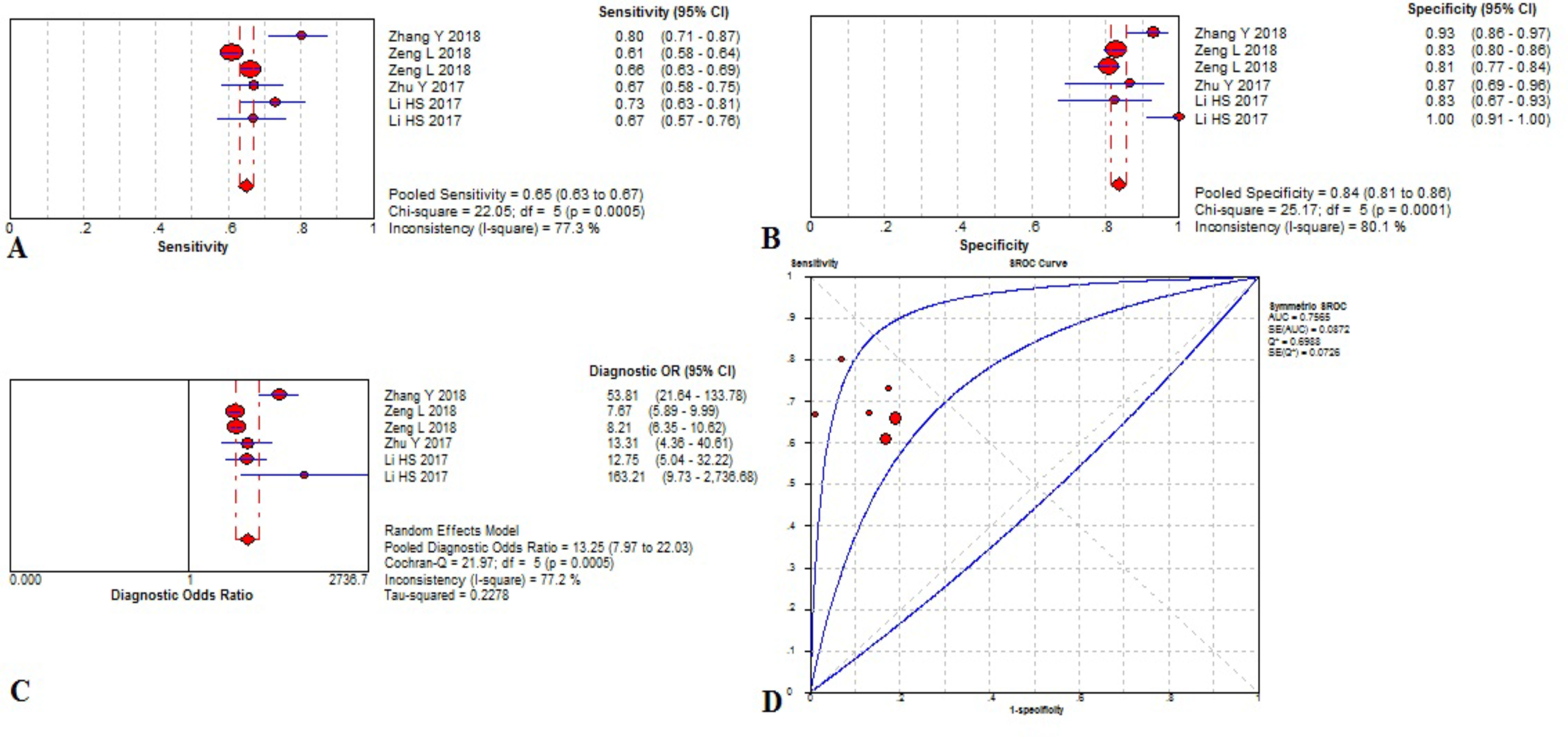

Supplement: S8 Fig — (A) Pooled sensitivity. (B) Pooled specificity. (C) Overall DOR. (D) The SROC curves for all data sets. The point estimates from each study are shown as solid squares. The pooled estimates are shown as a solid diamond. Effect sizes were pooled by random-effects models. Each square in the SROC curve represents one study. Sample size is indicated by the size of the square. Error bars represent 95% CIs. CI, confidence interval; miR, mircoRNA; SROC; summary receiver operating characteristic curves value; OR, odds ratio. (TIF) [file pone.0217523.s010.tif]
